# Dissecting Cellular Function and Distribution of β-Glucosidases in Trichoderma reesei

**DOI:** 10.1128/mBio.03671-20

**Published:** 2021-05-11

**Authors:** Ai-Ping Pang, Haiyan Wang, Yongsheng Luo, Zihuayuan Yang, Zhiyu Liu, Zhao Wang, Bingzhi Li, Song Yang, Zhihua Zhou, Xiaolin Lu, Fu-Gen Wu, Zuhong Lu, Fengming Lin

**Affiliations:** a State Key Laboratory of Bioelectronics, School of Biological Science and Medical Engineering, Southeast University, Nanjing, China; b Key Laboratory of Systems Bioengineering (Ministry of Education), School of Chemical Engineering and Technology, Tianjin University, Tianjin, China; c Shandong Province Key Laboratory of Applied Mycology, and Qingdao International Center on Microbes Utilizing Biogas, School of Life Sciences, Qingdao Agricultural University, Qingdao, China; d Key Laboratory of Synthetic Biology, Institute of Plant Physiology and Ecology, Shanghai Institutes for Biological Sciences, Chinese Academy of Sciences, Shanghai, China; University of Georgia

**Keywords:** filamentous fungi, *Trichoderma reesei*, cellulase, β-glucosidase, nonclassical secretory route, BFA-insensitive

## Abstract

Trichoderma reesei has 11 putative β-glucosidases in its genome, playing key parts in the induction and production of cellulase. Nevertheless, the reason why the *T. reesei* genome encodes so many β-glucosidases and the distinct role each β-glucosidase plays in cellulase production remain unknown. In the present study, the cellular function and distribution of 10 known β-glucosidases (CEL3B, CEL3E, CEL3F, CEL3H, CEL3J, CEL1A, CEL3C, CEL1B, CEL3G, and CEL3D) were explored in *T. reesei*, leaving out BGL1 (CEL3A), which has been well investigated. We found that the overexpression of *cel3b* or *cel3g* significantly enhanced extracellular β-glucosidase production, whereas the overexpression of *cel1b* severely inhibited cellulase production by cellulose, resulting in nearly no growth of *T. reesei*. Four types of cellular distribution patterns were observed for β-glucosidases in *T. reesei*: (i) CEL3B, CEL3E, CEL3F, and CEL3G forming clearly separated protein secretion vesicles in the cytoplasm; (ii) CEL3H and CEL3J diffusing the whole endomembrane as well as the cell membrane with protein aggregation, like a reticular network; (iii) CEL1A and CEL3D in vacuoles; (iv) and CEL3C in the nucleus. β-glucosidases CEL1A, CEL3B, CEL3E, CEL3F, CEL3G, CEL3H, and CEL3J were identified as extracellular, CEL3C and CEL3D as intracellular, and CEL1B as unknown. The extracellular β-glucosidases CEL3B, CEL3E, CEL3F, CEL3H, and CEL3G were secreted through a tip-directed conventional secretion pathway, and CEL1A, via a vacuole-mediated pathway that was achieved without any signal peptide, while CEL3J was secreted via an unconventional protein pathway bypassing the endoplasmic reticulum (ER) and Golgi.

## INTRODUCTION

β-glucosidases (BGL, E.C. 3.2.1.21) hydrolyze glycosidic bonds in oligosaccharides and glycosides to release nonreducing terminal glucosyl residues (1). They exist in all domains of living organisms, playing a variety of essential functions, including biomass conversion in microorganisms; cell wall lignification, cell wall β-glucan turnover, phytohormone activation, aromatic compound release, and defense in plants; and metabolism of glycolipids and exogenous glycosides in animals. These functions allow β-glucosidases to find wide applications in agricultural and industrial fields (2). As a major component of cellulase produced by fungi (*Penicllium*, Aspergillus, and *Trichoderma*) for biomass degradation in industry, β-glucosidases hydrolyze cellobiose and oligosaccharides released from insoluble cellulose by endoglucanase (EG; EC 3.2.1.4) and cellobiohydrolase (CBH; EC 3.2.1.91) into fermentable d-glucose that can be further utilized by microorganisms to produce biofuels, fine chemicals, and medicines. β-glucosidases have been an object of major research efforts, due to their important roles in the induction and production of cellulase (3–9).

Trichoderma reesei (teleomorph Hypocrea jecorina) is a major industrial strain for cellulase production due to its superior secretion ability (10–13). In total, 11 putative genes encoding β-glucosidases are predicted in the genome of *T. reesei* (14). They are grouped into glycoside hydrolase (GH) families 1 (*cel1a* and *cel1b*) and 3 (*cel3a*, *cel3b*, *cel3c*, *cel3d*, *cel3e*, *cel3f*, *cel3g*, *cel3h*, and *cel3j*) based on their sequence identity and structural similarity, which are archived in the CAZy (carbohydrate active enzymes) database (15, 16). These β-glucosidases are located at different genomic sites. Genes *cel3e*, *cel3f*, and *cel3g* are located in chromosome I, *cel1b* in chromosome II, *cel3a*, *cel1a*, and *cel3d* in chromosome III, *cel3h*, *cel3j*, and *cel3c* in chromosome IV, and *cel3b* in chromosome VI. They are not clustered in the genome and do not locate near chromosome ends (17). CEL3A, contributing to most of the extracellular β-glucosidase activity (18), was the first well-characterized β-glucosidase, which was localized throughout the whole fungal cell with enormous accumulation at the hyphal apexes (19). Deletion of *cel3a* led to a lag in the total extracellular protein level and the endoglucanase production in *T. reesei*, suggesting its essential role in rapid induction of cellulase production (20). Overexpression of *cel3a* in *T. reesei* has been utilized to successfully increase the extracellular β-glucosidase production (21, 22), and even the total cellulase production (23, 24). CEL1A and CEL1B were considered to be intracellular β-glucosidases and also played an important role in the rapid induction of cellulase (25, 26). Recently, it was reported that deletion of *cel3g* significantly improved cellulase production on lactose due to increased transcription of lactose permease that might be involved in lactose transport (14). Despite these research efforts on *T. reesei* β-glucosidases, the reason why the *T. reesei* genome encodes so many β-glucosidases and the distinct role each β-glucosidase plays in cellulase production remain unknown.

Secretory proteins of filamentous fungi are mainly released into the extracellular space using the classical secretory pathway. These proteins usually carry a signal peptide to direct their translocation into the lumen of the endoplasmic reticulum (ER) for protein folding, from where they reach the Golgi apparatus via coat protein complex II (COPII)-coated vesicles for further protein modification and then the plasma membrane for protein release. Filamentous fungi exhibit highly polarized growth with protein secretion occurring at the hyphal tips, where ER and Golgi apparatus are concentrated in a polarized manner (27). Non-tip-directed protein release is also observed at subapical hyphal compartments such as fungi septa (28) and lateral plasma membrane (29). What is more, some extracellular proteins can be secreted without entering the conventional secretion pathway, which is called unconventional protein secretion. Four different types of unconventional protein secretion have been uncovered—type I, pore-mediated translocation across the plasma membrane; type II, ABC transporter-based secretion; type III, autophagy-based secretion; and type IV, Golgi-bypass pathway to the plasma membrane (30, 31). Most unconventional secretion pathways are stimulated by stress that may compromise the functional integrity of the classical secretory pathway and trigger the demand for effective alternatives. Secreted proteins utilizing unconventional protein secretion pathways have been found in fungi, such as chitin synthases of Neurospora crassa (32), a cellobiase of the agaric fungus Termitomyces clypeatus (33), effector proteins during biotrophic invasion of Magnaporthe oryzae (27), and endochitinase Cts1 of Ustilago maydis (34). It has been suggested that cellulase excretion in *T. reesei* might be assisted through unconventional protein secretion pathways, though mostly it occurs via the conventional ER-Golgi secretion pathway (19). Nevertheless, our knowledge of the molecular basis of the nonclassical secretion process in filamentous fungi is still limited.

In this study, 10 recombinant *T. reesei* strains, Rcel3B, Rcel3E, Rcel3F, Rcel3H, Rcel3J, Rcel1A, Rcel3C, Rcel1B, Rcel3G, and Rcel3D, were successfully constructed by fusing red fluorescent protein (DsRed) to the C terminus of β-glucosidases CEL3B, CEL3E, CEL3F, CEL3H, CEL3J, CEL1A, CEL3C, CEL1B, CEL3G, and CEL3D, respectively, in *T. reesei* RUT-C30. Using these recombinant strains, the impact of each β-glucosidase overexpression on *T. reesei* cellulase production was investigated. The cellular distributions of the 10 β-glucosidases were monitored in living hyphae of recombinant *T. reesei* strains. Moreover, extracellular β-glucosidases were identified by detecting the fluorescence kinetics in the supernatant of the mutants with a fluorescence microplate reader, whose secretion pathways were explored.

## RESULTS

### The effect of overexpression of β-glucosidase on cellulase production.

**FIG 1:**
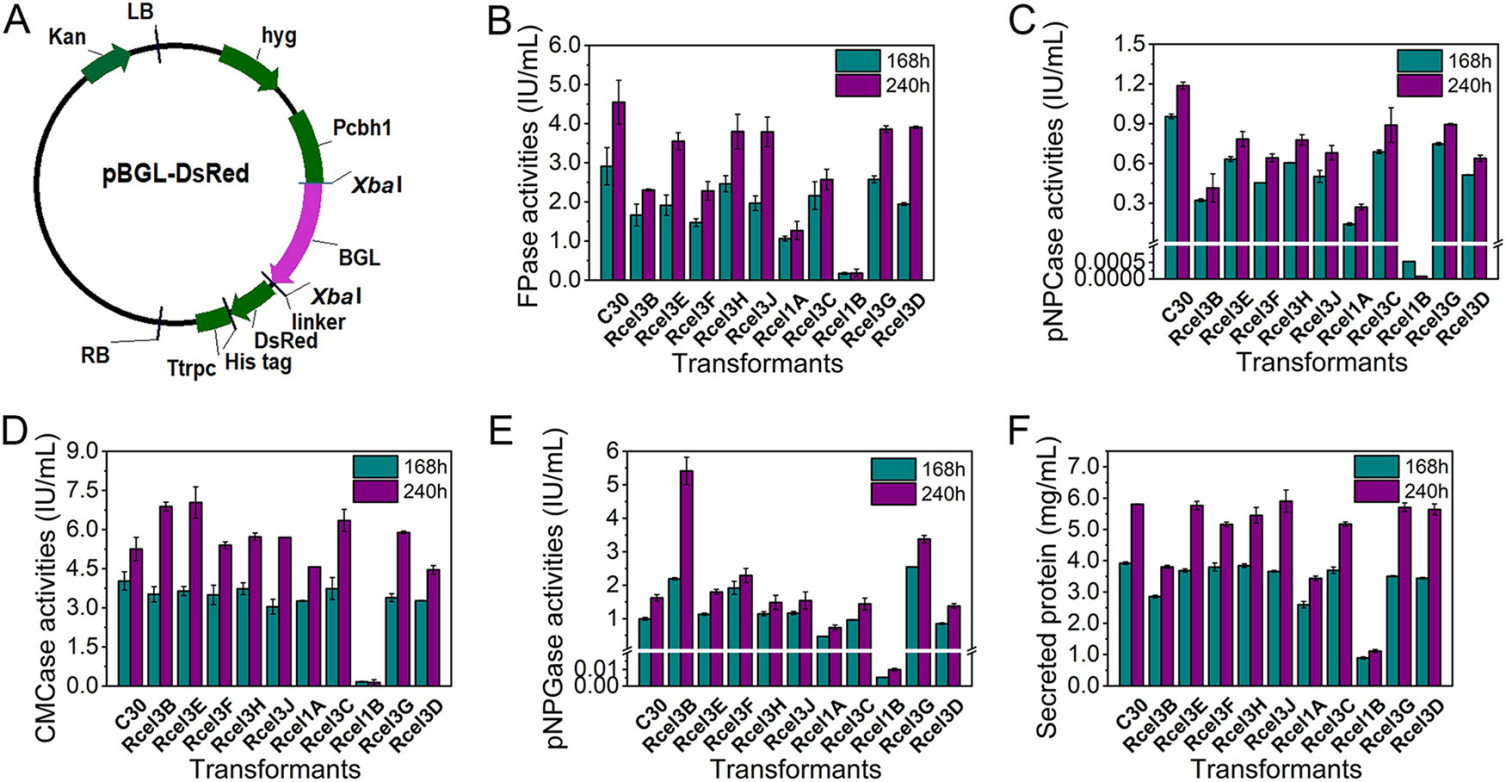
Cellulase activities of recombinant *T. reesei* strains cultured in TMM plus 2% cellulose for 168 h and 240 h. (A) Schematic illustration of the plasmid pBGL-DsRed (BGL = CEL3B, CEL3E, CEL3F, CEL3H, CEL3J, CEL1A, CEL3C, CEL1B, CEL3G, or CEL3D). Kan, kanamycin resistance; LB, left border of the binary vector; RB, right border of the binary vector; Pcbh1, a modified CBH1 promoter; Ttrpc, Aspergillus nidulans trpC terminator; linker, a short sequence linked β-glucosidase encoding gene and DsRed; hyg, hygromycin B phosphotransferase gene. (B) The FPase activity: the filter paper activity. (C) The pNPCase activity: the CBH activity. (D) The CMCase activity: the CMC activity. (E) The pNPGase activity: the β-glucosidase activity. (F) Protein concentration. Data are represented as the mean of three independent experiments, and error bars express the standard deviations.

Expression of β-glucosidase CEL3B, CEL3E, CEL3F, CEL3H, CEL3J, CEL1A, CEL3C, CEL1B, CEL3G, or CEL3D as a fusion protein with red fluorescence protein DsRed was individually performed in *T. reesei* RUT-C30 (Fig. 1A), generating recombinant strains Rcel3B, Rcel3E, Rcel3F, Rcel3H, Rcel3J, Rcel1A, Rcel3C, Rcel1B, Rcel3G, and Rcel3D, respectively. For each recombinant strain, we randomly selected 2 transformants for cellulase activity assay and red fluorescence observation by culturing them in *Trichoderma* minimal medium (TMM) with 2% cellulose. We found that the two transformants of each recombinant strain showed similar cellular distributions, pNPGase activity (the β-glucosidase activity), and FPase activity (the filter paper activity) (Fig. S1). Thus, one transformant for each BGL was selected for further study.

10.1128/mBio.03671-20.2 FIG S1 (A and B) Confocal images (A) and cellulase activities (B) of recombinant *T. reesei* strains cultured in TMM plus 2% cellulose for 168 h. In particular, the confocal images for Rcel1B were taken at 240 h. The pNPGase activity—the β-glucosidase activity; the FPase activity—the filter paper activity. Data are represented as the mean of three independent experiments, and error bars express the standard deviations. Download FIG S1, JPG file, 2.6 MB. Copyright © 2021 Pang et al. 2021 Pang et al. https://creativecommons.org/licenses/by/4.0/ This content is distributed under the terms of the Creative Commons Attribution 4.0 International license.

The effect of the expression of these β-glucosidases on cellulase production of *T. reesei* using cellulose as the carbon source was investigated by comparison with the parent strain *T. reesei* RUT-C30 (Fig. 1B to F). Both the FPase activity (Fig. 1B) and the pNPCase activity (the CBH activity) (Fig. 1C) were reduced in all the recombinant strains. For strains Rcel3B, Rcel3E, Rcel3F, Rcel3H, Rcel3J, Rcel3C, and Rcel3G, the CMCase activity (the CMC activity) was lower than that of RUT-C30 at 168 h but rose quickly in the following 72 h to the same level as or a higher level than that of RUT-C30 at 240 h, demonstrating delayed endoglucanase production in these *T. reesei* transformants (Fig. 1D). The pNPGase activity at 168 h was increased by 121%, 93%, and 156% in strains Rcel3B, Rcel3F, and Rcel3G, respectively, was decreased by 52.7%, 99.4% and 14.5% in strains Rcel1A, Rcel1B, and Rcel3D, respectively, and was almost unchanged for Rcel3C, Rcel3E, Rcel3H, and Rcel3J (Fig. 1E). Specifically, the pNPGase activity in strains Rcel3B and Rcel3G was notably increased by 2.33- and 1.08-fold, respectively, at 240 h, demonstrating that the overexpression of *cel3b* and *cel3g* can significantly increase the β-glucosidase production, which was also observed for *cel3a* (21, 22). The total protein concentration of the supernatant stayed almost unchanged in strains Rcel3E, Rcel3F, Rcel3H, Rcel3J, Rcel3C, Rcel3G, and Rcel3D but decreased in Rcel3B, Rcel1A, and Rcel1B (Fig. 1F). It is worth noting that all the cellulase activities and the total extracellular protein concentration were decreased sharply in strain Rcel1B, followed by strain Rcel1A. It seems that the overexpression of CEL1B-DsRed in *T. reesei* inhibits the cellulase production severely.

### Characterization of the recombinant *T. reesei* strains.

**FIG 2:**
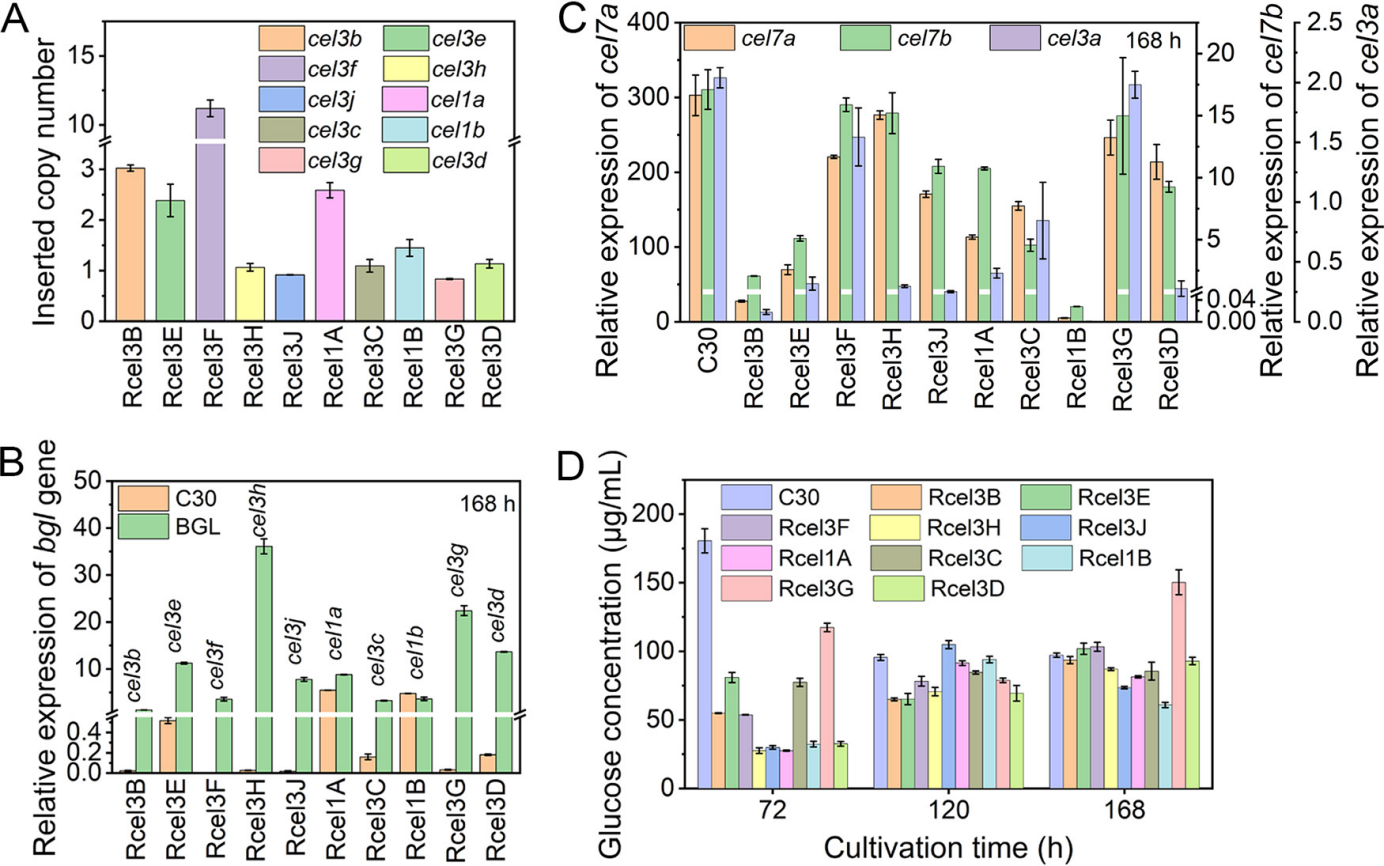
(A to D) qPCR analysis of the copy numbers (A), the transcription levels of each β-glucosidase gene (B), three major cellulase genes, *cel7a*, *cel7b*, and *cel3a* at 168 h (C), and glucose concentration (D) in *T. reesei* RUT-C30 and the recombinant strains. Data are represented as the mean of three independent experiments, and error bars express the standard deviations.

The inserted copy number and the mRNA level of each β-glucosidase gene in the corresponding recombinant strain were measured by quantitative PCR (qPCR) (Fig. 2). The inserted copy number was 1 for genes *cel3h*, *cel3j*, *cel3c*, *cel1b*, *cel3g*, and *cel3d*, 2 or 3 for *cel3e* and *cel1a*, 3 for *cel3b*, and 11 for *cel3f* (Fig. 2A). Correspondingly, the mRNA levels of genes *cel3b*, *cel3e*, *cel3f*, *cel3h*, *cel3j*, *cel1a*, *cel3c*, *cel1b*, *cel3g*, and *cel3d* in the corresponding recombinant strains were 1.3, 11.2, 3.6, 36.1, 7.8, 8.8, 3.3, 3.6, 22.4, and 13.6, respectively (Fig. 2B). The mRNA levels of genes *cel3b*, *cel3e*, *cel3f*, *cel3h*, *cel3j*, *cel3c*, *cel3g*, and *cel3d* were increased remarkably compared to RUT-C30. In contrast, the mRNA levels of genes *cel1a* and *cel1b* stayed almost unchanged in strains Rcel1A and Rcel1B at 168 h, but were increased by 53.3% and 55.4%, respectively, at 120 h (Fig. S2). Furthermore, the transcriptional levels of cellulase genes encoding cellobiohydrolase CBH1 (CEL7A), endoglucanase CMC (CEL7B), and β-glucosidase BGL1 (CEL3A) were reduced along with the overexpression of *cel3b*, *cel3e*, *cel3j*, *cel3c*, and *cel3d*, while they stayed constant with the overexpression of *cel3f* and *cel3g* (Fig. 2C). In strains Rcel1A and Rcel1B, even though the mRNA levels of genes *cel1a* and *cel1b* were not changed markedly, the transcriptional levels of cellulase genes CBH1, CMC, and BGL1 were decreased notably. In strain Rcel3H, the overexpression of *cel3h* severely inhibited the expression of β-glucosidase BGL1 (Fig. 2B) but caused no noticeable change in the expression of CBH1 and CMC (Fig. 2C). Though only one copy of *cel3h* was inserted, strain Rcel3H displayed the highest mRNA level of *cel3h* (Fig. 2C). The glucose concentrations in the fermentation supernatant of each recombinant strain were also checked (Fig. 2D). The extracellular glucose concentration was reduced significantly in the recombinant strains at 72 h, which was not that significant at 120 h and 168 h. Obviously, the overexpression of each β-glucosidase gene decreased the cellulase production at the transcription level (Fig. 2C). The decreased cellulase production was not due to central carbon catabolite repression (CCR), as glucose in the supernatants of the recombinant strains was not increased compared to that of RUT-C30 (Fig. 2D).

10.1128/mBio.03671-20.3 FIG S2 The transcription levels of the genes *cel1a* and *cel1b* at 120 h in *T. reesei* strains RUT-C30, Rcel1A, and Rcel1B. Download FIG S2, JPG file, 0.6 MB. Copyright © 2021 Pang et al. 2021 Pang et al. https://creativecommons.org/licenses/by/4.0/ This content is distributed under the terms of the Creative Commons Attribution 4.0 International license.

### Cellular distribution of β-glucosidases.

**FIG 3:**
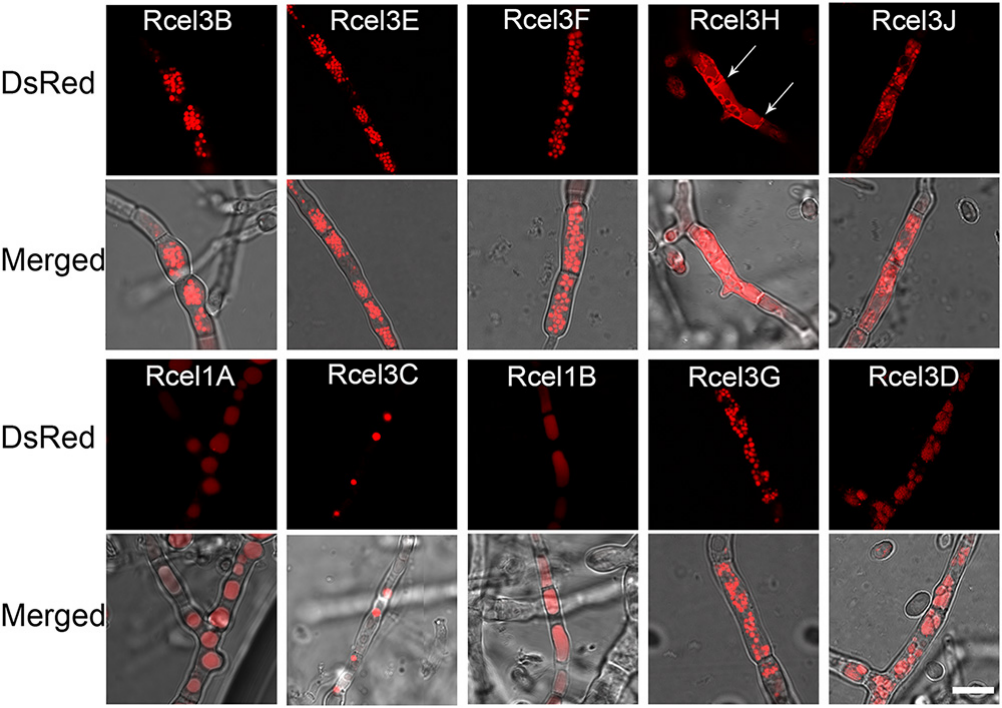
Cellular distribution of CEL3B-DsRed, CEL3E-DsRed, CEL3F-DsRed, CEL3H-DsRed, CEL3J-DsRed, CEL1A-DsRed, CEL3C-DsRed, CEL1B-DsRed, CEL3G-DsRed, and CEL3D-DsRed in strains Rcel3B, Rcel3E, Rcel3F, Rcel3H, Rcel3J, Rcel1A, Rcel3C, Rcel1B, Rcel3G, and Rcel3D, respectively. The confocal images were observed under CLSM at 120 h for all strains except strains Rcel1B and Rcel3D, which were monitored at 216 h and 168 h, respectively. All the strains were grown in TMM plus 2% cellulose. The white arrow indicates the septa of Rcel3H. Scale bar = 10 μm.

The cellular distribution of different β-glucosidases was observed by confocal laser scanning microscopy (CLSM) using the recombinant strains cultivated in TMM plus 2% cellulose (Fig. 3 and Fig. S3). Large sums of dense red fluorescent punctuates, probably secretion vesicles, were found throughout the cytoplasm of strains Rcel3B, Rcel3E, Rcel3F, and Rcel3G, while no fluorescence was observed on the cell membrane/wall of these strains. In strains Rcel3H and Rcel3J, red fluorescence was observed in a reticular network with brighter nodes. In these two strains, the red fluorescence was diffused on the endomembrane and the cell membrane/wall. In particular, the red fluorescence was also found in the septa of strain Rcel3H. In strain Rcel3C, a series of red fluorescent dots were distributed in the cytoplasm, whose granular fluorescent pattern was totally different from that found in strains Rcel3B, Rcel3E, Rcel3F, and Rcel3G but similar to that of the reported nuclear proteins in *T. reesei*, such as CLP1 (35), Rce1 (36), MAT1-2-1 (37), XYR1 (38), and CRE1 (38). This result suggests that the protein CEL3C-DsRed is possibly in the nucleus. In strain Rcel1A, globular fluorescent bodies were found, which were much larger than those of strains Rcel3B, Rcel3E, Rcel3F, Rcel3G, and Rcel3C, indicating that CEL1A-DsRed was probably located in vacuoles. CEL3D-DsRed was also mostly located in vacuoles with protein aggregation, unlike that from CEL1A-DsRed diffusing throughout the whole vacuoles.

10.1128/mBio.03671-20.4 FIG S3 Confocal images of CEL3B-DsRed, CEL3E-DsRed, CEL3F-DsRed, CEL3H-DsRed, CEL3J-DsRed, CEL1A-DsRed, CEL3C-DsRed, CEL1B-DsRed, CEL3G-DsRed, and CEL3D-DsRed in strains Rcel3B, Rcel3E, Rcel3F, Rcel3H, Rcel3J, Rcel1A, Rcel3C, Rcel1B, Rcel3G, and Rcel3D, respectively. The confocal images were observed under CLSM at 120 h for all strains except strains Rcel1B and Rcel3D, which were monitored at 216 h and 168 h, respectively. All the strains were grown in TMM plus 2% cellulose. Scale bar = 10 μm. Download FIG S3, JPG file, 1.7 MB. Copyright © 2021 Pang et al. 2021 Pang et al. https://creativecommons.org/licenses/by/4.0/ This content is distributed under the terms of the Creative Commons Attribution 4.0 International license.

The red fluorescence appeared at 48 h in strains Rcel3B, Rcel3E, Rcel3F, Rcel3H, Rcel1A, and Rcel3G, 72 h in Rcel3J and Rcel3C, 96 h in Rcel3D, and 216 h in Rcel1B (Fig. S4). The cellulase production was almost abolished in strain Rcel1B (Fig. 1), leading to the extremely poor growth of strain Rcel1B on cellulose. Therefore, the red fluorescence of protein CEL1B-DsRed was not observed until 216 h, which was very weak.

10.1128/mBio.03671-20.5 FIG S4 Confocal images of recombinant *T. reesei* strains with the expression level of the corresponding β-glucosidases at the indicated time points in the figures. (A) Rcel3B; (B) Rcel3E; (C) Rcel3F; (D) Rcel3H; (E) Rcel3J; (F) Rcel1A; (G) Rcel3C; (H) Rcel1B; (I) Rcel3G; and (J) Rcel3D. Scale bar = 10 μm. Download FIG S4, JPG file, 0.4 MB. Copyright © 2021 Pang et al. 2021 Pang et al. https://creativecommons.org/licenses/by/4.0/ This content is distributed under the terms of the Creative Commons Attribution 4.0 International license.

To correlate the timing at which fluorescence appears to the timing of the expression level of each β-glucosidase, the mRNA level of each β-glucosidase gene at the time when fluorescence appears and 24 h before the fluorescence appearance was measured (Fig. S4). Overexpression of *cel3b*, *cel3e*, *cel3f*, *cel3j*, *cel3c*, or *cel3d* was observed in the respective recombinant strains at both time points. Overexpression of *cel3h* and *cel1a* was found only at the time of fluorescence appearance, while overexpression of *cel3g* was observed only at 24 h before the fluorescence appearance. It seems that there is no direct connection between intracellular fluorescence intensity and the transcriptional level of each β-glucosidase (Fig. S4). For instance, although the mRNA level of *cel3c* in strain Rcel3C was extremely higher than that of *cel3b* in strain Rcel3B at 48 h, its fluorescence intensity was weaker than that of *cel3b*.

### Identification of extracellular β-glucosidases.

**FIG 4:**
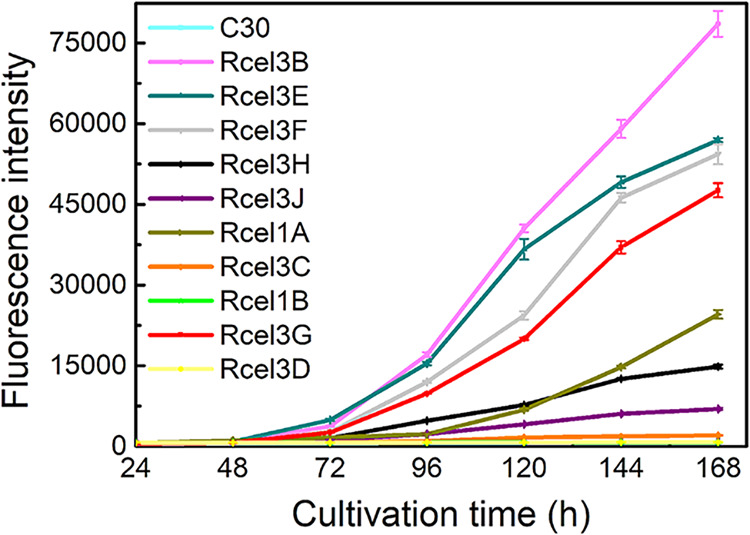
Fluorescence intensity of the supernatants from the parent strain RUT-C30 and 10 recombinant strains, Rcel3B, Rcel3E, Rcel3F, Rcel3H, Rcel3J, Rcel1A, Rcel3C, Rcel1B, Rcel3G, and Rcel3D cultured in TMM plus 2% cellulose for 168 h. Data are represented as the mean of three independent experiments, and error bars express the standard deviations.

To identify whether the tested β-glucosidases are intracellular or extracellular, real-time monitoring of fluorescence intensity in fermentation supernatant was performed for all the recombinant strains and the parent strain RUT-C30 using a fluorescence microplate reader (Fig. 4). No fluorescence was detected in the supernatant of RUT-C30 and its recombinant strain that expressed DsRed alone (19). The fluorescence intensity in the supernatant of strains Rcel1B, Rcel3C, and Rcel3D was similar to that of RUT-C30 and almost stayed unchanged during the whole fermentation process, which suggested that proteins CEL1B-DsRed, CEL3C-DsRed, and CEL3D-DsRed were not secreted into the culture medium. Therefore, CEL3C and CEL3D are intracellular proteins. However, the expression of CEL1B-DsRed was too weak to determine whether CEL1B was intracellular or extracellular, due to almost no cell growth of strain Rcel1B on cellulose. In contrast, the fluorescence intensity in the supernatant of the other seven recombinant strains, Rcel3B, Rcel3E, Rcel3F, Rcel3H, Rcel3J, Rcel1A, and Rcel3G, was increased gradually along with the fermentation time, reaching 109-, 79-, 76-, 21-, 10-, 34-, and 55-fold that of RUT-C30 at 168 h, respectively. This suggested that all these seven DsRed-labeled β-glucosidases were secreted into the extracellular space, indicating that they are extracellular. Except for CEL1A-DsRed, the dynamic fluorescence intensity of these extracellular β-glucosidases had little change at the first 48 h, began to rise in the next 24 h, and went up rapidly from 72 h to 168 h during the cultivation process, showing a similar trend of protein secretion as that of CEL7A (CBH) and CEL7B (CMC) (19). The fluorescence intensity of CEL1A-DsRed was not remarkably increased until 96 h, similar to that of the major extracellular β-glucosidase CEL3A (19). This suggested differences in effective secretion time of these extracellular β-glucosidases. In this way, we showed that β-glucosidases CEL3B, CEL3E, CEL3F, CEL3H, CEL3J, CEL1A, and CEL3G are extracellular, while CEL3C and CEL3D are intracellular. According to their fluorescence intensity in the supernatant, the extracellular β-glucosidases were classified into two groups, the high-secretion (CEL3B, CEL3E, CEL3F, and CEL3G) and the intermediate-secretion (CEL3H, CEL3J, and CEL1A) groups.

### The extracellular β-glucosidases CEL3B, CEL3E, CEL3F, CEL3H, and CEL3G were secreted through tip-directed protein trafficking pathways, while CEL3J and CEL1A were secreted through non-tip-directed pathways.

**FIG 5:**
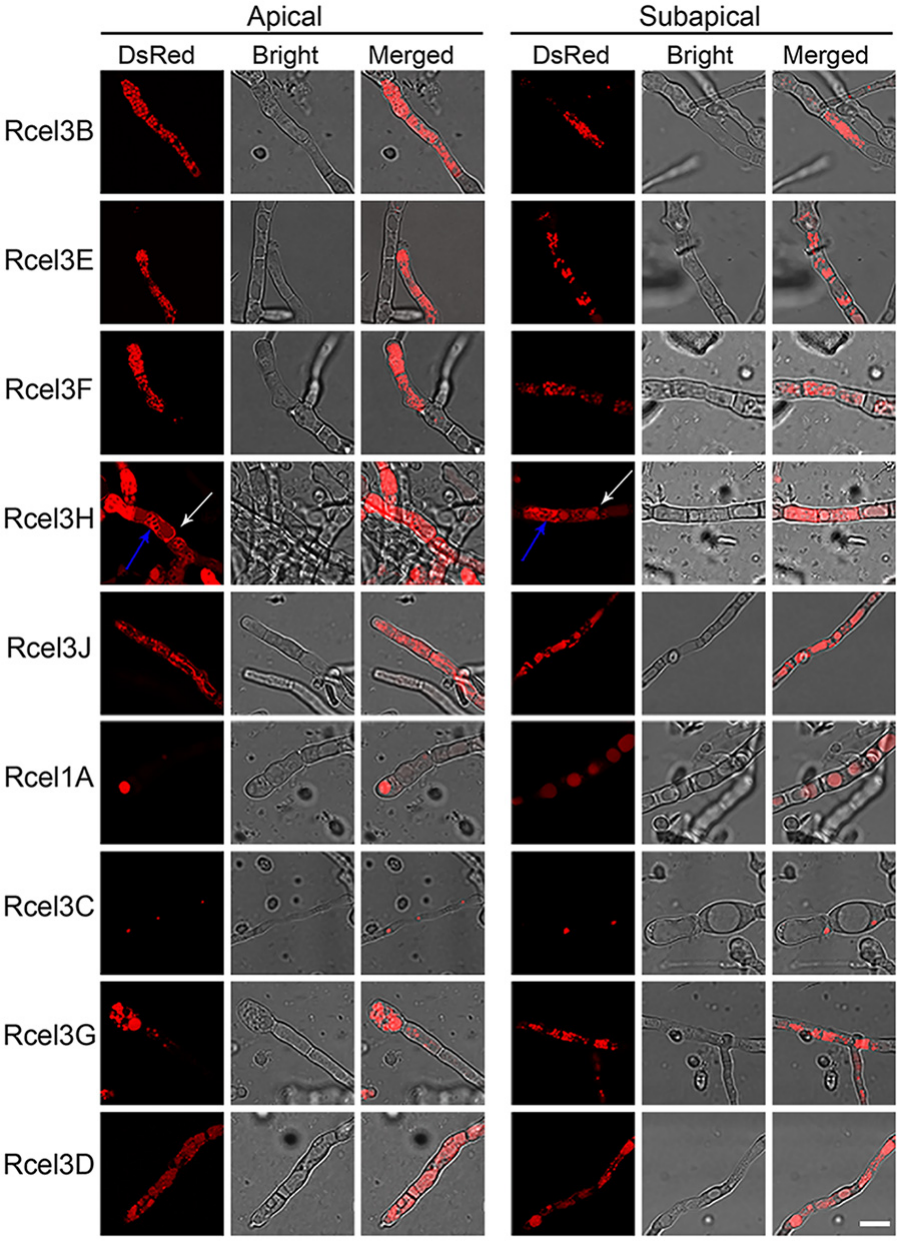
Cellular localizations of CEL3B-DsRed, CEL3E-DsRed, CEL3F-DsRed, CEL3H-DsRed, CEL3J-DsRed, CEL1A-DsRed, CEL3C-DsRed, CEL3G-DsRed, and CEL3D-DsRed at the apical and subapical regions of nine recombinant strains, Rcel3B, Rcel3E, Rcel3F, Rcel3H, Rcel3J, Rcel1A, Rcel3C, Rcel3G, and Rcel3D, respectively. The confocal images were observed under CLSM at 120 h for all strains except Rcel3D, which was monitored at 168 h. The white and blue arrows indicate the septa and organelle membranes of Rcel3H, respectively. All the strains were grown in TMM plus 2% cellulose. Scale bar = 10 μm.

A detailed cellular distribution of subapical and apical compartments of the identified extracellular β-glucosidases in *T. reesei* mutants was imaged to investigate their individual protein trafficking pathways. The proteins CEL3B-DsRed, CEL3E-DsRed, CEL3F-DsRed, CEL3H-DsRed, and CEL3G-DsRed distributed along the hyphal tube in gradient-like fashion, with higher concentrations at apical regions (Fig. 5 and Fig. S5). This indicated that these five β-glucosidases were secreted to the extracellular space via tip-directed vesicle trafficking, which was the same as CEL3A (19). Among these five β-glucosidases, only CEL3H-DsRed was located abundantly on the cell membrane/wall, septa, and organelle membranes (Fig. 5), implying that the secretion of CEL3H-DsRed might be assisted by both tip- and non-tip-directed protein trafficking pathways. The other two extracellular β-glucosidases, CEL3J-DsRed and CEL1A-DsRed, did not show gradient fluorescent distributions from the apex to the subapex, indicating that their transport might occur via non-tip-directed secretion pathways. Formation of vacuoles occurred in both the apical and subapical segments of the hyphae in strain Rcel1A.

10.1128/mBio.03671-20.6 FIG S5 Confocal images and line-scanning fluorescence intensity profiles of the marked regions (the white arrow) of CEL3B-DsRed, CEL3E-DsRed, CEL3F-DsRed, CEL3H-DsRed, and CEL3G-DsRed in recombinant strains Rcel3B, Rcel3E, Rcel3F, Rcel3H, and Rcel3G, respectively, which were observed under CLSM at 120 h. Scale bar = 10 μm. Download FIG S5, JPG file, 0.3 MB. Copyright © 2021 Pang et al. 2021 Pang et al. https://creativecommons.org/licenses/by/4.0/ This content is distributed under the terms of the Creative Commons Attribution 4.0 International license.

### The extracellular β-glucosidases CEL3B, CEL3E, CEL3F, CEL3H, CEL1A, and CEL3G entered the ER, while CEL3J did not.

**FIG 6:**
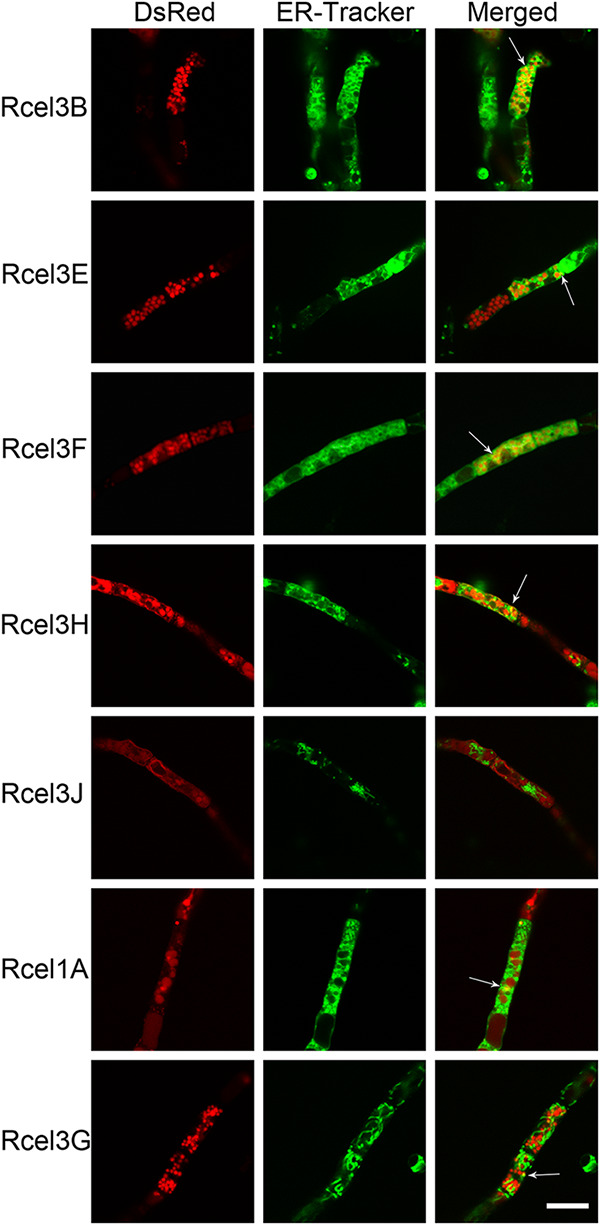
Confocal images of strains Rcel3B, Rcel3E, Rcel3F, Rcel3H, Rcel3J, Rcel1A, and Rcel3G stained with ER-Tracker. The white arrow indicates the overlap of DsRed-labeled β-glucosidases with ER-Tracker. Scale bar = 10 μm.

ER-tracker with green fluorescence was utilized to stain the seven strains Rcel3B, Rcel3E, Rcel3F, Rcel3H, Rcel3J, Rcel1A, and Rcel3G to determine if extracellular β-glucosidases enter the ER for protein modification and processing. Except for strain Rcel3J, there was a partial overlap of green and red fluorescence in the other six strains, leading to yellow fluorescence as pointed out by the white arrows in Fig. 6 and Fig. S6, indicative of the colocalization of the six DsRed-labeled β-glucosidases with the ER. This result showed clearly that CEL3B, CEL3E, CEL3F, CEL3H, CEL1A, and CEL3G entered the ER, while CEL3J did not. Interestingly, the green fluorescence of Rcel3J was much weaker than that of the other recombinant strains, demonstrating that the functional integrity of the ER was compromised severely in strain Rcel3J.

10.1128/mBio.03671-20.7 FIG S6 Confocal images of mutant strains Rcel3B, Rcel3E, Rcel3F, Rcel3H, Rcel3J, Rcel1A, and Rcel3G stained with ER-Tracker. The white arrow indicates the overlap of DsRed-labelled β-glucosidases with ER-Tracker. Scale bar = 20 μm. Download FIG S6, JPG file, 0.7 MB. Copyright © 2021 Pang et al. 2021 Pang et al. https://creativecommons.org/licenses/by/4.0/ This content is distributed under the terms of the Creative Commons Attribution 4.0 International license.

### Cellular distribution of β-glucosidases under native conditions.

**FIG 7:**
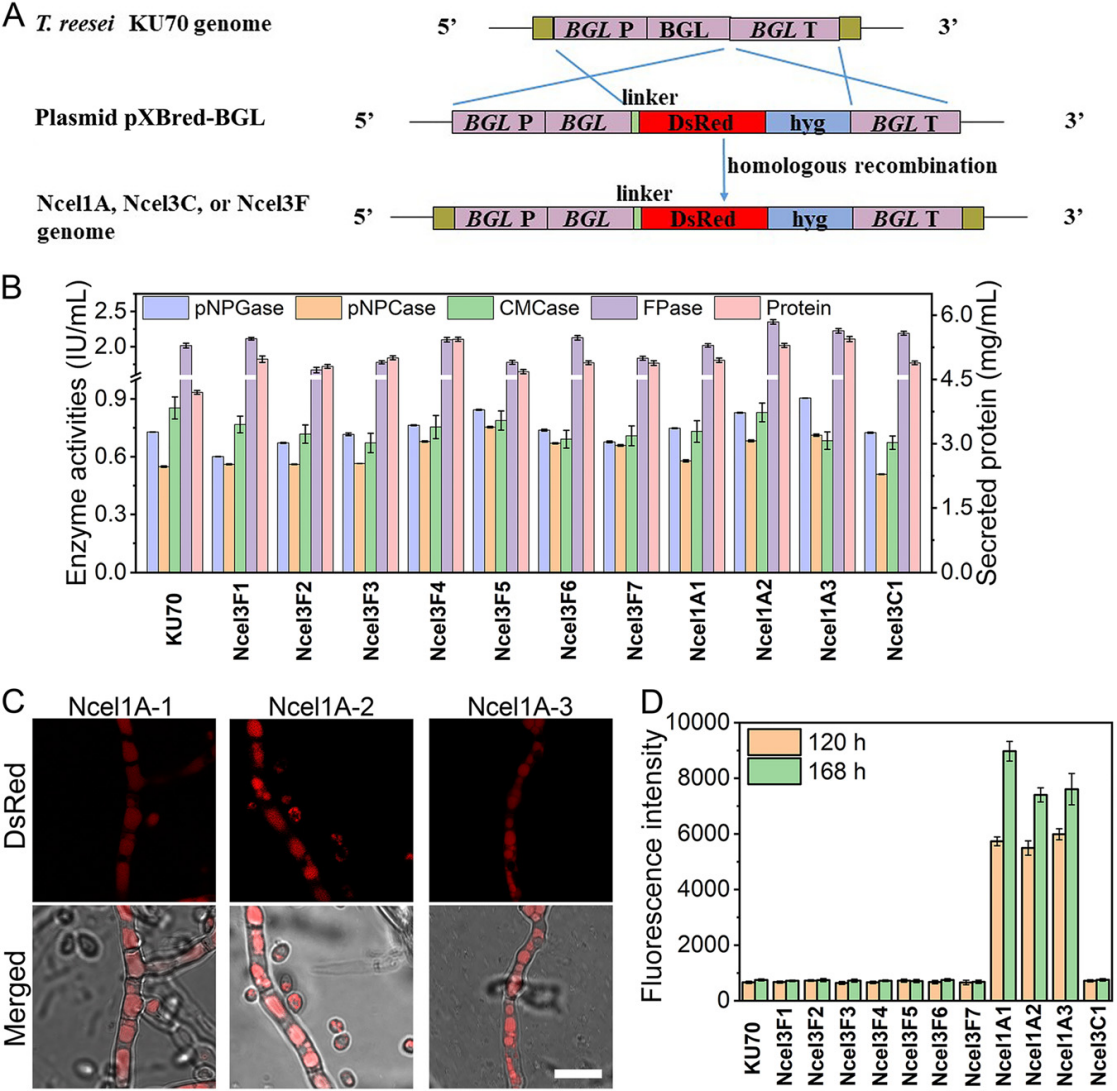
(A) Schematic illustration of plasmid construction for labeling BGLs (CEL1A, CEL3C, and CEL3F) under native conditions. *BGL* P, *BGL* promoter; *BGL*, *BGL* coding sequence; *BGL* T, *BGL* terminator; linker, a short sequence linking β-glucosidase and DsRed; hyg, hygromycin B phosphotransferase. (B) Cellulase activities and secreted protein at 168 h. (C) Cellular distributions of strains Ncel1A1-3 at 120 h. (D) Fluorescence intensity of culture supernatant at 120 h and 168 h. All *T. reesei* strains, KU70, Ncel1A1-3, Ncel3C1, and Ncel3F1-7, were cultured in TMM plus 2% cellulose. Data are represented as the mean of three independent experiments, and error bars express the standard deviations.

To prove that the observed β-glucosidase localizations in BGL-DsRed overexpressed strains reflect those of the native β-glucosidases, we further studied the cellular distribution of a couple of β-glucosidases under the control of their own promoter by integrating fluorescence gene *DsRed* at their endogenous locus. On the basis of their transcriptional levels in *T. reesei* RUT-C30 (Fig. 2B), the 10 studied β-glucosidases can be categorized into three groups—*cel1a* and *cel1b* with a relative mRNA level of >1 to the housekeeping gene *sar1* (39); *cel3e*, *cel3d*, and *cel3c* with a 0.1 < relative mRNA level of <1; and *cel3g*, *cel3h*, *cel3b*, *cel3j*, and *cel3f* with a relative mRNA level of <0.1. Therefore, *cel1a*, *cel3c*, and *cel3f*, representing each group of β-glucosidases, respectively, were selected for cellular distribution investigation under native conditions. To achieve this, plasmids for fusing fluorescence gene *DsRed* to each β-glucosidase at its own endogenous locus through homologous recombination were constructed and transformed individually into *T. reesei* KU70 (Fig. 7). KU70 was obtained from RUT-C30 by deleting gene *ku70* for efficient homologous recombination (40) (Fig. 7A). We obtained 3, 1, and 7 transformants for *cel1a*, *cel3c*, and *cel3f*, respectively, which were named Ncel1A1-3, Ncel3C1, and Ncel3F1-7. The cellulase activities and the secreted protein in these recombinant strains Ncel1A1-3, Ncel3C1, and Ncel3F1-7 changed little compared with the parental strain *T. reesei* KU70, demonstrating that the integration of fluorescence gene *DsRed* did not affect the cellulase activities. Cellular observation by CLSM showed that the red fluorescence in strains Ncel1A1-3 appeared at 48 h and was located mainly in vacuole (Fig. 7C), which was the same as that observed in strain Rcel1A (Fig. 3). The fluorescence intensity of culture supernatant in Ncel1A was increased along with time (Fig. 7D), suggesting that CEL1A could be secreted to the extracellular space. Therefore, CEL1A was proved to be extracellular and distributed in vacuole independent of random overexpression of CEL1A-DsRed or integration of DsRed at the endogenous locus of CEL1A, suggesting that the observed localizations under CEL1A overexpression conditions reflect those of the native CEL1A. Unfortunately, for strains Ncel3C1 and Ncel3F1-7, red fluorescence could not be detected either intracellularly or extracellularly during a 7-day fermentation process, possibly due to the low transcriptional level of *cel3c* or *cel3f* (Fig. 2B). It seems that *cel3c* and *cel3f* must be overexpressed to study their cellular distribution.

### BFA inhibited cellulase secretion in Rcel3B, Rcel3E, Rcel3F, Rcel3H, Rcel1A, and Rcel3G, but not Rcel3J.

**FIG 8:**
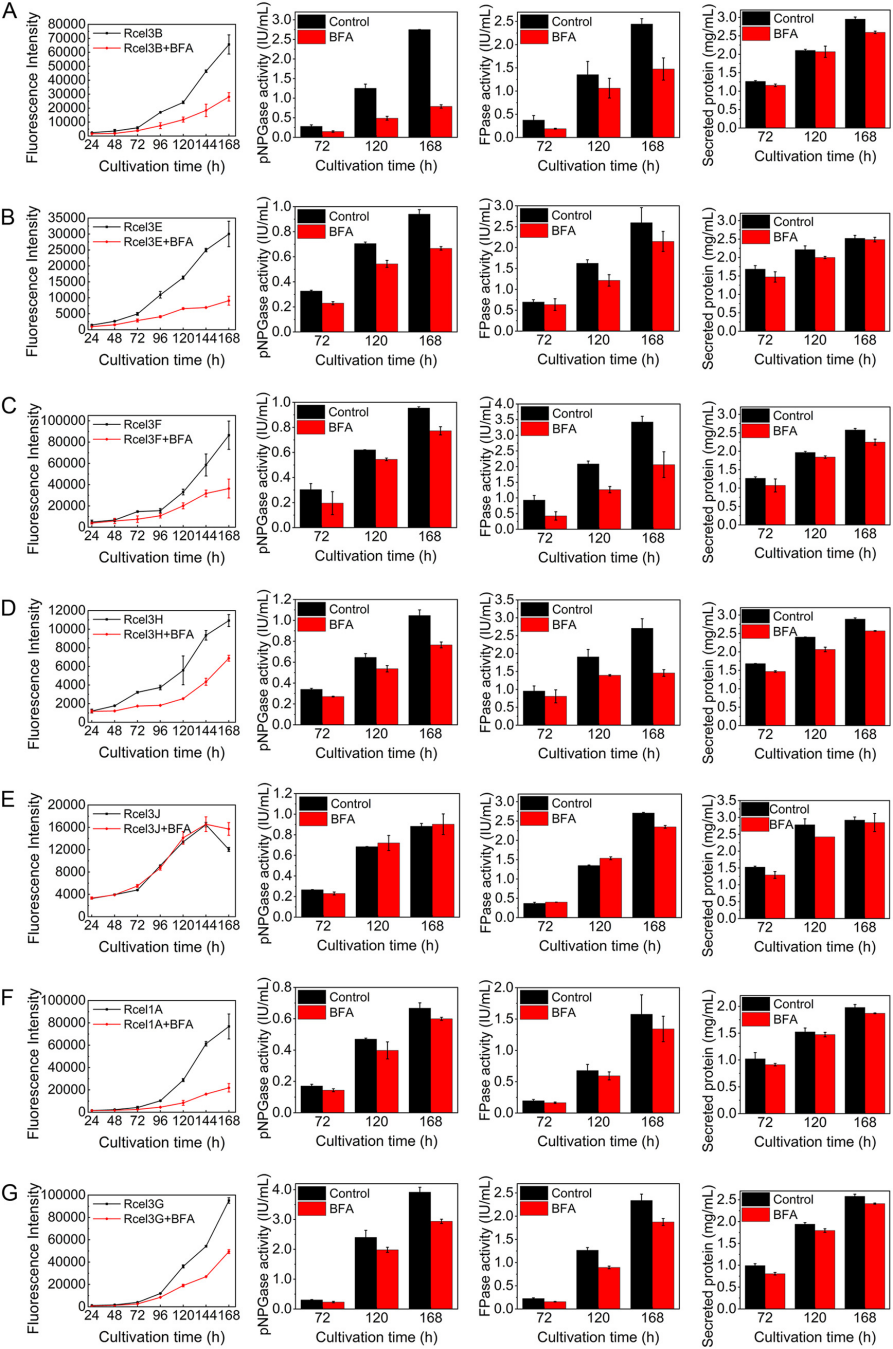
(A to G) The effect of BFA on cellulase secretion for strains Rcel3B (A), Rcel3E (B), Rcel3F (C), Rcel3H (D), Rcel3J (E), Rcel1A (F), and Rcel3G (G). All the strains were cultured in TMM plus 2% cellulose with/without 10 μg/ml BFA for 168 h. Data are represented as the mean of three independent experiments and error bars express the standard deviations.

To further study the secretion pathways of the seven extracellular β-glucosidases, the corresponding mutant strains were treated with 10 μg/ml brefeldin A (BFA), which interferes with Golgi body-dependent trafficking (32, 41). As indicated by the unchanged red fluorescence in the mycelia of all BFA-treated strains compared to the untreated ones (Fig. S7), the expression of each DsRed-tagged β-glucosidase in the corresponding recombinant strain was not affected by BFA at the tested concentration, similar to that of DsRed-tagged CMC, CBH, and CEL3A (19). However, the fluorescence intensity of the supernatant showed different characteristics among the BFA-treated strains (Fig. 8). For strains Rcel3B, Rcel3E, Rcel3F, Rcel3H, Rcel1A, and Rcel3G, the fluorescence intensity in the supernatant was decreased notably in the presence of BFA throughout the whole fermentation process (Fig. 8A to D, F, and G), suggesting significantly reduced secretion of these extracellular β-glucosidases. Meanwhile, the pNPGase activity, the FPase activity, and secreted protein concentration were also decreased to various degrees in these recombinant strains treated with BFA (Fig. 8A to D, F, and G). Interestingly, the fluorescence intensity in the supernatant of strain Rcel3J was not affected by the addition of BFA (Fig. 8E), which implied that CEL3J secretion was insensitive to BFA. It seems that the secretion of CEL3J was via an alternative secretion pathway bypassing the Golgi. The more interesting thing is that the pNPGase activity in the BFA-treated strain Rcel3J was similar to that of the untreated strains. This result suggests that the major extracellular β-glucosidase production was also not sensitive to BFA at all in Rcel3J, showing different secretion behavior from that in strains Rcel3B, Rcel3E, Rcel3F, Rcel3H, Rcel1A, RBGL (19), RCMC (19), and RCBH (19). It seems that the expression of CEL3J-DsRed might impart BFA resistance to the cellulase secretion in *T. reesei*.

10.1128/mBio.03671-20.8 FIG S7 Confocal images of recombinant strains Rcel3B, Rcel3E, Rcel3F, Rcel3H, Rcel3J, Rcel1A, and Rcel3G cultured in TMM plus 2% cellulose with/without 10 μg/ml BFA at 120 h. Scale bar = 10 μm. Download FIG S7, JPG file, 0.06 MB. Copyright © 2021 Pang et al. 2021 Pang et al. https://creativecommons.org/licenses/by/4.0/ This content is distributed under the terms of the Creative Commons Attribution 4.0 International license.

### CEL3H and CEL3J were located on the cell membrane but not the cell wall.

**FIG 9:**
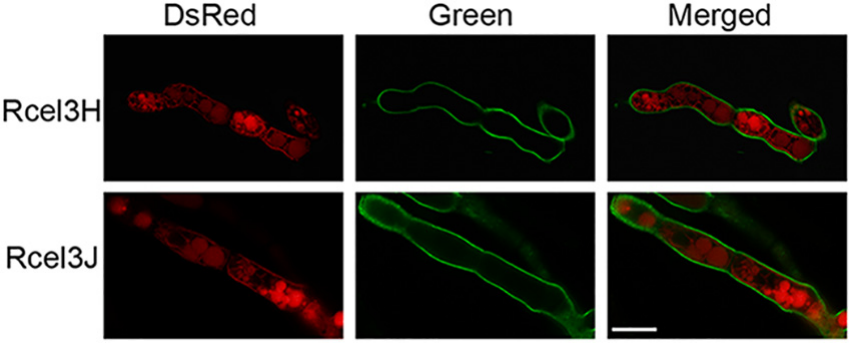
Confocal images of strains Rcel3H and Rcel3J stained with GC-PEG-cholesterol-FITC, a green fluorescence dye for cell walls. Scale bar = 10 μm.

Our result indicates that both CEL3H and CEL3J were probably localized on the cell membrane/wall (Fig. 2 and Fig. 5). To determine whether they are on cell membrane or cell wall, a green fluorescent dye, GC-PEG-cholesterol-FITC, for cell wall imaging was employed (42). As shown in Fig. 9, green fluorescence was observed on the cell wall of Rcel3H and Rcel3J, which was not overlapped with the red fluorescence of either CEL3H-DsRed or CEL3J-DsRed, as demonstrated by a lack of yellow fluorescence in the merged figures. This result suggests that CEL3H and CEL3J were distributed on cell membrane rather than cell wall.

### CEL3C was a nuclear protein.

**FIG 10:**
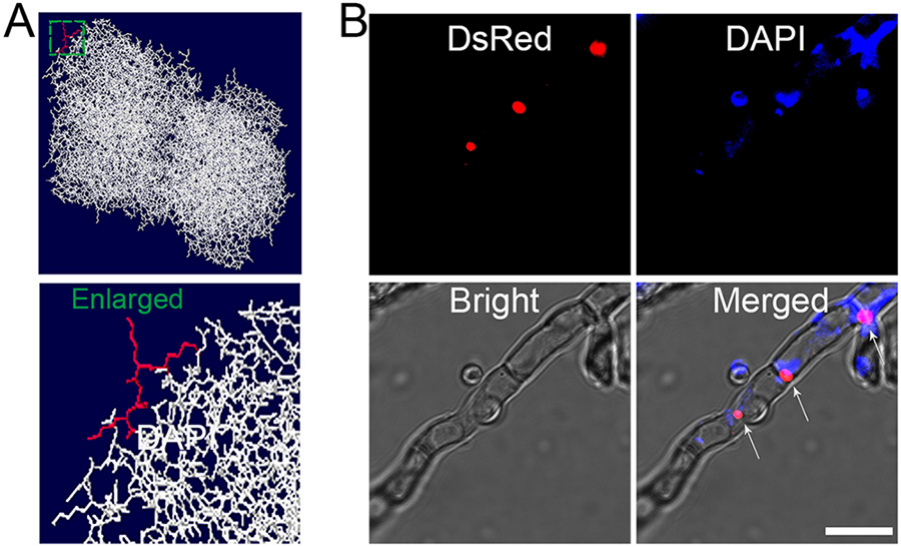
Localization of CEL3C in *T. reesei* Rcel3C. (A) Homology modeling of CEL3C using SWISS-MODEL. The amino acids KKKK highlighted in red were on the surface of the 3D structure of CEL3C. The area marked with the green square, which contains the amino acids KKKK, was enlarged and presented. (B) Confocal images of Rcel3C stained with the nuclear dye DAPI. Scale bar = 10 μm.

It is worth noting that CEL3C-DsRed was distributed as a series of red dots in strain Rcel3C (Fig. 5) as found for other nuclear proteins in *T. reesei*, such as CLP1 (35), Rce1 (36), MAT1-2-1 (37), XYR1 (38), and CRE1 (38), suggesting its possible localization in the cell nucleus. The import of nuclear protein into the cell nucleus is mediated by the nuclear localization signal (NLS) that has a basic amino acid cluster rich in lysine or arginine. CEL3C contained NLS (^324^VLPLSKKKKL^334^) as predicted by NLS Mapper (http://nls-mapper.iab.keio.ac.jp/cgi-bin/NLS_Mapper_form.cgi). Protein homology modeling of CEL3C on SWISS-MODEL (http://swissmodel.expasy.org/) revealed that the basic amino acid residues KKKK in the NLS were exposed to the surface of the three-dimensional (3D) structure of CEL3C (Fig. 10A), benefiting from nuclear import. The cell nucleus of Rcel3C was stained blue by the nuclear dye DAPI (4′,6-diamidino-2-phenylindole), which was colocalized with the red fluorescence of CEL3C-DsRed, generating pink fluorescence in the merged picture (Fig. 10B). This further shows that CEL3C was targeted to the nucleus of hyphal cells.

## DISCUSSION

**FIG 11:**
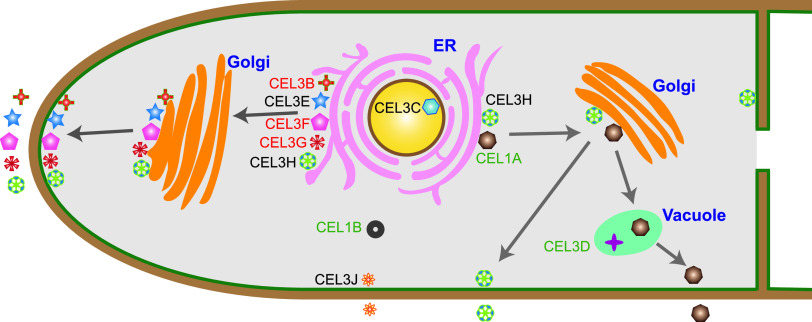
Cellular distribution and protein secretion pathways of β-glucosidases CEL3B, CEL3E, CEL3F, CEL3H, CEL3J, CEL1A, CEL3C, CEL1B, CEL3G, and CEL3D in *T. reesei*. CEL3B, CEL3E, CEL3F, CEL3H, CEL3J, CEL1A, and CEL3G were extracellular β-glucosidases. CEL3B, CEL3E, CEL3F, and CEL3G were secreted through the tip-directed conventional ER-Golgi secretion pathway. CEL3H was located on the cell membrane and endomembrane and was secreted through tip- and non-tip-directed conventional ER-Golgi secretion pathways. CEL1A was in vacuole and secreted via the vacuole-mediated conventional ER-Golgi secretion pathway. CEL3J was distributed on the cell membrane and endomembrane and secreted via an unconventional protein pathway bypassing the ER and Golgi. The intracellular β-glucosidases CEL3C and CEL3D were in the nucleus and vacuole, respectively. CEL1B was observed in the cytoplasm with extremely low expression. The names of β-glucosidases are marked with different colors according to the impact of their overexpression on cellulase expression, which are categorized into three types—CEL1A, CEL1B, and CEL3D, marked in green, whose overexpression repressed all the cellulase activities, including pNPGase, pNPCase, and CMCase; CEL3B, CEL3F, and CEL3G, marked in red, whose overexpression decreased the pNPCase activity, increased the pNPGase activity, and did not reduce the CMCase activity; and CEL3C, CEL3E, CEL3H, and CEL3J, highlighted in black, whose overexpression decreased the pNPCase activity, and left the pNPGase activity unchanged, without compromising the CMCase activity.

Early studies have demonstrated that β-glucosidases are involved in the regulation of cellulase biosynthesis in *T. reesei*, affecting the expression and secretion of cellulase both in amounts and in speed (20, 21, 25), which is further supported by our findings here. The endoglucanase production was delayed in all the recombinant strains expressing different DsRed-tagged β-glucosidases, except for strains Rcel1A, Rcel1B, and Rcel3D, while the pNPCase activity was decreased in all the recombinant strains. Based on the different impacts on overexpression of different cellulase components, the 10 investigated β-glucosidases were classified into three types (Fig. 11)—CEL1A, CEL1B, and CEL3D, whose overexpression repressed all the cellulase activities, including pNPGase, pNPCase, and CMCase; CEL3B, CEL3F, and CEL3G, whose overexpression decreased the pNPCase activity, increased the pNPGase activity, and did not reduce the CMCase activity; and CEL3C, CEL3E, CEL3H, and CEL3J highlighted in black in Fig. 11, whose overexpression decreased the pNPCase activity and left the pNPGase activity unchanged, without compromising the CMCase activity. The overexpression of BGL genes *cel1a*, *cel1b*, and *cel3d* decreased the pNPGase activity (Fig. 1E), which might lead to cellobiose accumulation. This, in turn, could cause product inhibition of upstream cellobiohydrolase CBH and endoglucanase CMC, repressing both the transcription levels and the protein levels of CBH1 and CMC (Fig. 2C, Fig. 1C and D), thus slowing down the cellulose hydrolysis (43, 44). In contrast to the reduced pNPCase activity, the CMCase activity was increased at 240 h in strains Rcel3B, Rcel3E, and Rcel3C in spite of a delay, indicating that the overexpression of β-glucosidases affected different cellulase components in different ways. It seems that the different cellulase components can be regulated separately through unknown intricate mechanisms, which requires more study. Therefore, further study on when and how cellulase components can be tuned separately might be an interesting research direction to reveal the complicated fungal regulatory networks for fine-tuning the expression and secretion of cellulase (45, 46).

The overexpression of *cel3b* and *cel3g* remarkably increased extracellular β-glucosidase production, similar to *cel3a* (19). A neighbor-joining phylogram of the putative β-glucosidases revealed that CEL3B and CEL3G shared high protein sequence homology to the major extracellular β-glucosidase CEL3A (47). Proteins CEL3A and CEL3B showed similar catalytic properties, both of which exhibited high specific activity against cello-oligosaccharides and were capable of catalyzing transglycosylation reactions from cellobiose, indicating that CEL3B is probably an alternative to CEL3A. In contrast, CEL3G only showed high substrate specificity toward pNPG. Unlike CEL3A (19), CEL3B and CEL3G were not on the cell membrane.

On the other hand, the overexpression of *cel1b* completely hampered the induction of cellulase production by cellulose, resulting in almost no growth of *T. reesei*. This was consistent with early findings that the simultaneous absence of *cel1a* and *cel1b* resulted in a marked increase of the extracellular β-glucosidase activity at 12 h (25). More interestingly, gene *cel1b* is predicted to contain 6 putative binding sites for the carbon catabolic repressor CRE1 that represses cellulase production (48). Nevertheless, the expression of *cel1b* was increased in *T. reesei* Δ*cre1* with high cellulase production and was downregulated in both *T. reesei* Δ*xyr1* (48) and ZC121 (49) with fairly low cellulase production on cellulose. In addition, it was found that knockout of gene *cel1b* in strain TU-6 (the uridine-auxotrophic derivative of *T. reesei* QM9414) caused only a slight decrease of intracellular pNPGase activity on cellulose (25). The contradictory findings from these previous studies and our study might be explained by the fact that the appropriate level of *cel1b* expression is required for efficient cellulase production. Also, the overexpression of CEL1A-DsRed reduced the cellulase production moderately in *T. reesei* on cellulose. Previous research showed that the CMCase, pNPCase, and FPase activity on day 7 have little change growing on cellulose when *cel1a* was deleted in QM9414 (50). It was found that knockout of gene *cel1a* or *cel1b* did not affect the expression of cellulase but delayed the expression of cellulase (25). *cel1a* and *cel1b* are the highly induced genes in *T. reesei* growing on cellulose or sophorose (51), whose encoded proteins exhibited significant transglycosylation activity when incubated with relatively high concentrations of glucose and cellobiose (25).

Based on the confocal imaging result by CLSM, the cellular distribution patterns of β-glucosidases can be divided into four classes: (i) CEL3B, CEL3E, CEL3F, and CEL3G, forming clearly separated protein secretion vesicles in the cytoplasm; (ii) CEL3H and CEL3J, diffusing the whole endomembrane as well as cell membrane with protein aggregation, like a reticular network; (iii) CEL1A and CEL3D in vacuoles; and (iv) CEL3C in the nucleus. The type II cellular distribution is reminiscent of the major cellulase components CEL3A, CBH, and CMC (19).

Among the 10 tested β-glucosidases, CEL3B, CEL3E, CEL3F, CEL3H, CEL3J, CEL1A, and CEL3G were identified as extracellular, CEL3C and CEL3D as intracellular, and CEL1B as unknown. According to the signal peptide in the N-terminal sequences of the protein (http://www.cbs.dtu.dk/services/SignalP/) (52), β-glucosidases CEL3B, CEL3E, CEL3F, CEL3H, and CEL3G are predicted to be extracellular, which was confirmed by our result. Nevertheless, both CEL3G and CEL1A were reported to be intracellular in some early studies (14, 53). When overexpressed under the constitutive Ppdc promoter or the inducing Pcbh1 and Pxyn2 promoters, the level of CEL3G was low and was not detected in the fermentation supernatant by Western blotting (14). In contrast, when the constitutive promoter Ptef1 was utilized for its overexpression, the expression of CEL3G was very high and was found to be extracellular (14). The protein level of CEL3G was good both in cytoplasm and in the supernatant as observed by CLSM (Fig. 3) and a microplate reader, respectively (Fig. 4). It is probably the case that the lack of secretion in the previous study was due to its low protein concentration in *T. reesei*. CEL1A has also been reported to be intracellular by Western blotting (53). This inconsistency might be due to the relatively poor secretion level of CEL1A at the early fermentation stage as demonstrated by the low fluorescence intensity of CEL1A-DsRed on day 3 (Fig. 4), leading to the unsuccessful detection of the extracellular CEL1A on day 3 by Western blotting as performed in the literature (53). In addition, the *cel1a* homologue, *bgl4* from Humicola grisea, which shared 73.1% identity, was also proved to be extracellular (54). Therefore, we believe that CEL3G and CEL1A are extracellular β-glucosidases. As shown here, it is more reliable to investigate whether protein is extracellular or intracellular by monitoring the dynamic fluorescence intensity during the whole cultivation process. CEL1B does not have the signal peptide for protein secretion and was previously reported to be intracellular (47), which could not be confirmed in our study owing to the fact that only very weak red fluorescence was observed in strain Rcel1B at the end of fermentation (216 h).

Although CEL1A and CEL3J do not contain the signal peptide for protein secretion, we do find them to be extracellular (Fig. 4), indicating that these two proteins are secreted by the unconventional secretion pathways (31). However, CEL1A-DsRed was partially overlapped with ER (Fig. 6), and its secretion was notably inhibited in the presence of BFA (Fig. 8). Moreover, CEL1A was distributed mainly in the vacuoles without enrichment in the apical region (Fig. 5). It seems that CEL1A containing no classical signal peptide was transported to the extracellular medium via the conventional protein pathway involving the ER and Golgi apparatus, which was vacuole-mediated rather than tip-directed. There is a hypothesis that the Golgi-dependent vacuole-mediated secretory route exits in filamentous fungi for regulatory protein secretion (55–57). In this nontip conventional pathway, proteins are secreted via the fusion of vacuoles with lateral plasma membrane in mature hyphal segments. Similarly, BGL-DsRed, CBH-DsRed, and CMC-DsRed were also observed to reside in the lumen of globular vacuoles (19) in addition to xylanase II (XYN II) (58) and endoglucanase I (59). It is supposed that the cellulase secretion by *T. reesei* might involve vacuoles (58, 60). These hydrolytic enzymes are stored in the vacuoles in a poorly active form to avoid the deleterious effect of their hyperproduction in *T. reesei* (60). However, unlike BGL, CBH, and CMC, CEL1A does not contain any signal peptide for secretion. How CEL1A can enter the ER without signal peptide is unknown. The leaderless proteins such as fibroblast growth factor 9 (Fgf-9) (61) and an oxidoreductase (POR) from Cryphonectria parasitica (62) have been reported to enter the ER and traverse the Golgi complex in a similar manner to other constitutively secreted proteins, even though they do not contain signal peptide, a transmembrane region, or Golgi localization information (62).

CEL3J was localized to the cell membrane (Fig. 9) and was also extracellular (Fig. 4). It could reach the plasma membrane via an unconventional protein pathway that did not involve the ER and Golgi apparatus (Fig. 11), as demonstrated by its lack of colocalization with ER tracker (Fig. 6) and resistance to BFA (Fig. 8). What is more, the total β-glucosidase secretion in strain Rcel3J was also BFA-insensitive. Although the expression level of *cel3j* in strain Rcel3J was much higher than that in RUT-C30, it was still lower than that of *cel3e* in Rcel3E, *cel3h* in Rcel3H, *cel1a* in Rcel1A, *cel3g* in Rcel3G, and *cel3d* in Rcel3D (Fig. 2B). However, only the overexpression of *cel3j* affected ER integrity in strain Rcel3J, whose protein secretion was insensitive to BFA via bypassing the ER. Based on this, we do not think these results regarding an alternate secretion pathway of CEL3J were due to the overexpression of *cel3j*. A similar phenomenon has been reported in *Termitomyces clypeatus*, where the cellobiase titer was increased by 17% in the presence of 50 μg/ml BFA, although the total protein secretion was inhibited by 30%. It is suggested that an alternative secretory pathway was operative to deliver cellobiase to the vacuoles for secretion without passing the Golgi (33). In another study, the secretion of the cytoplasmic effectors in the rice blast fungus Magnaporthe oryzae was BFA-resistant, suggesting their secretion is via the unconventional Golgi-independent pathway (63). The secretion of these cytoplasmic effectors is facilitated via the biotrophic interfacial complex, which has not been identified in *T. reesei*.

It is unexpected to find that one of the tested β-glucosidases was located in the nucleus, namely, CEL3C. Nevertheless, the role of CEL3C is still unknown. Both CEL3C and CEL3D are intracellular, having XYR1-binding sites in the promoter regions (64). They were low in abundance and may have been somewhat more highly induced by cellulose than by sophorose in QM6a (51). They behaved like aryl β-glucosidases, showing high preference for pNPG (47). Nevertheless, unlike CEL3C, CEL3D was mainly located in vacuoles with protein aggregation. It seems that some of the 11 β-glucosidases in *T. reesei* are not mainly for cellulose degradation/cellulase production. For a better understanding of the function of these β-glucosidases, it is worthwhile to explore how the recombinant strains overexpressing each β-glucosidase behave under other conditions, such as noncellulose carbon sources such as lactose, glucose, galactose, and glycerol, which is undergoing extensive study in our lab.

It is worth noting that the protein localization of β-glucosidases was studied by overexpression of their fusion with DsRed in this study. Sometimes, the protein overexpression might complicate the interpretation of the results when the overexpression of proteins saturates intracellular transport mechanisms, leading to abnormal subcellular localization. However, *T. reesei* possesses efficient protein synthesis and protein secretion ability (11–13). Though all the tested overexpressed β-glucosidases in their respective recombinant strains did not replicate normal expression, their transcription levels were no more than 12% of that of CBH1 in RUT-C30 (Fig. 2B). Thus, the overexpression of each β-glucosidase should not be a burden to the cells, avoiding abnormal distribution. Moreover, we have labeled CEL1A with fluorescence protein DsRed at its endogenous locus under its own promoter to prove that the cellular distribution of CEL1A-DsRed under overexpression conditions reflects that of the native CEL1A. Therefore, the cellular distribution of BGL-DsRed fusions that we observed was probably ascribed to each BGL itself fused with DsRed, but not gene overexpression. Early studies regarding protein localization have also used heterologous promoters. For example, heterologous promoters tcu1, CBH1, and CaMV-35S were used for investigating subcellular localization of nuclear proteins (CLP1 [35], Rce1 [36], MAT1-2-1 [37], XYR1 [38], and CRE1 [34]) in *T. reesei*, SNARE proteins (SNCI, SSOI, and SSOII) (29) in *T. reesei*, and a unique protein RxLR3 in Phytophthora brassicae (65), respectively. Another study about searching strains with anomalous temporal or spatial protein localization also used heterologous promoter xyl or van (66). Unfortunately, red fluorescence was not observed in strains Ncel3C1 and Ncel3F1-7, which might due to the low transcriptional level of *cel3c* and *cel3f*, making it impossible to investigate the cellular distribution of these two proteins under native conditions. For some proteins whose expression is too low to be detected under endogenous promoters, overexpression is required to investigate their protein localization (67, 68). Knowledge of cellular localization under overexpressed conditions has the potential under normal conditions to provide insights into the protein localization, giving direction for further investigation. Considering these findings together, we believe that the gene overexpression using promoter CBH1 could not interfere with our result on protein localization of β-glucosidases.

In conclusion, the effect of β-glucosidases on cellulase production was first investigated together with their spatiotemporal localization and secretion by successfully constructing recombinant *T. reesei* strains expressing red fluorescent protein DsRed-tagged versions of each β-glucosidase. The endoglucanase production was delayed in all the recombinant strains expressing different BGL-Dsred except strains Rcel1A, Rcel1B, and Rcel3D. The overexpression of *cel1b* almost repressed the cellulase production completely, while the overexpression of *cel3b* or *cel3g* significantly enhanced extracellular β-glucosidase production. The cellular distribution patterns of β-glucosidases were classified into (i) CEL3B, CEL3E, CEL3F, and CEL3G, displaying protein secretion vesicles in the cytoplasm; (ii) CEL3H and CEL3J, distributing through the whole endomembrane and cell membrane with protein aggregation, like a reticular network; (iii) CEL1A and CEL3D in vacuoles; and (iv) CEL3C in the nucleus. CEL3B, CEL3E, CEL3F, CEL3H, CEL3J, CEL1A, and CEL3G were identified as extracellular, CEL3C and CEL3D as intracellular, and CEL1B as unknown. The extracellular β-glucosidases CEL3B, CEL3E, CEL3F, CEL3H, and CEL3G were secreted through the tip-directed classical secretion pathway, and CEL1A was secreted through via the vacuole-mediated pathway that was achieved without any signal peptide, while CEL3J was secreted via a nonclassical secretion route independent of both ER and Golgi. These findings have implications for our fundamental understanding of β-glucosidases in filamentous fungi.

## MATERIALS AND METHODS

### Microbial strains and plasmids.

Escherichia coli DH5α was used as the cloning host (Vazyme Biotech Co., Ltd., Nanjing, China). *T. reesei* RUT-C30 (CICC 13052) was applied as a host for expressing DsRed-tagged β-glucosidases. Agrobacterium tumefaciens AGL-1 was utilized as a transfer DNA (T-DNA) donor for fungal transformation. A T-DNA binary plasmid, p-DsRed, containing a red fluorescent protein-coding gene was maintained in our laboratory and used to construct the transformation vectors (19).

### Construction of *T. reesei* mutants.

All the primers used in this study are listed in Table S1.

10.1128/mBio.03671-20.1 TABLE S1 Primers for β-glucosidase cloning and qPCR. Download Table S1, DOCX file, 0.02 MB. Copyright © 2021 Pang et al. 2021 Pang et al. https://creativecommons.org/licenses/by/4.0/ This content is distributed under the terms of the Creative Commons Attribution 4.0 International license.

For construction of BGL-DsRed-overexpressed strains, genes *cel3b*, *cel3e*, *cel3f*, *cel3h*, *cel3j*, *cel1a*, *cel3c*, *cel1b*, *cel3g*, and *cel3d* were amplified from the genomic DNA of *T. reesei* RUT-C30 and ligated to the plasmid p-DsRed at the XbaI site using the ClonExpress II one-step cloning kit (Vazyme Biotech Co., Ltd., Nanjing, China). The resulting expression vectors were transformed into RUT-C30 with an *Agrobacterium*-mediated fungal transformation (AMT) method using hygromycin B as a marker, yielding recombinant strains Rcel3B, Rcel3E, Rcel3F, Rcel3H, Rcel3J, Rcel1A, Rcel3C, Rcel1B, Rcel3G, and Rcel3D, respectively.

To label BGLs at their endogenous locus, we first ligated a short linker (GGTGGCGGTGGCTCGGGTGGCGGTGGCTCG) and DsRed to pXBthg (69) with the ClonExpress MultiS one-step cloning kit (Vazyme Biotech Co., Ltd., Nanjing, China), resulting in plasmid pXBred. Then the upstream (including the promoter and coding sequence) and downstream (including the terminator) fragments of *cel1a*, *cel3c*, or *cel3f* amplified from the genomic DNA of *T. reesei* RUT-C30 were ligated to the plasmid pXBred at the XhoI and BamHI sites, respectively. The resulting expression vectors were transformed into *T. reesei* KU70 (70) by the AMT method using hygromycin B as a marker, yielding recombinant strains Ncel1A, Ncel3C, and Ncel3F, respectively. The verification of DNA integration in the recombinant strains was confirmed by PCR (Fig. S8) and sequencing of the PCR product at Sangon Biotech.

10.1128/mBio.03671-20.9 FIG S8 (A) PCR confirmation of recombinant *T. reesei* strains Rcel3B, Rcel3E, Rcel3F, Rcel3H, Rcel3J, Rcel1A, Rcel3C, Rcel1B, Rcel3G, and Rcel3D and RUT-C30 (C30). (B) PCR confirmation of recombinant *T. reesei* strains Ncel3F1-7, Ncel1A1-3, Ncel3C1, and KU70. *T. reesei* RUT-C30 (C30) and KU70 were used as the negative control. Download FIG S8, JPG file, 0.1 MB. Copyright © 2021 Pang et al. 2021 Pang et al. https://creativecommons.org/licenses/by/4.0/ This content is distributed under the terms of the Creative Commons Attribution 4.0 International license.

### Cultivation of *T. reesei*.

The conidial suspension was grown in Sabouraud dextrose broth (SDB) at 28°C for 48 h. Then the culture was transferred into a 250-ml flask containing 50 ml TMM supplemented with 2% (wt/vol) cellulose and cultivated at 28°C. The TMM medium was as followed: tryptone, 0.75 g/liter; yeast extract, 0.25 g/liter; urea, 1.00 g/liter; (NH_4_)_2_SO_4_, 4.00 g/liter; KH_2_PO_4_, 6.59 g/liter; maleic acid, 11.6 g/liter; FeSO_4_ · 7H_2_O, 0.005 g/liter; MnSO_4_ · H_2_O, 0.0016 g/liter; ZnSO_4_ · 7H_2_O, 0.0014 g/liter; CoCl_2_ · 6H_2_O, 0.002 g/liter; MgSO_4_, 0.60 g/liter; CaCl_2_, 0.60 g/liter; Tween 80, 0.186 ml/liter (71). The pH of TMM was adjusted to 5.8 to 6.0 with NaOH. For the BFA experiment, 5.0 ml cultures, which were grown in TMM plus 2% cellulose for 4 days, were transferred into the same fresh TMM plus 2% cellulose supplemented with 10 μg/ml BFA and cultured for another 7 days. The 1.0-ml culture was sampled at different time points as indicated in the text.

### Analysis assays.

Samples were centrifuged at 8000 × *g* for 10 min to collect supernatants for cellulase activity and fluorescence intensity measurement. Cellulase activity assays were carried out as previously described (19, 21, 23, 49). Fluorescence intensity was determined at excitation/emission wavelengths of 540/635 nm under a fluorescence spectrophotometer (Hitachi Ltd., Japan). The glucose concentration was measured using a glucose detection kit (Shanghai Rongsheng Biotech, China).

### qRT-PCR analysis and copy number assay.

Fresh mycelia were harvested at the indicated time pints in the text. RNA extraction, reverse transcription, and reverse transcription-quantitative PCR (qRT-PCR) were performed as reported previously (19). The relative mRNA level was calculated by comparing to the housekeeping gene *sar1* (39). The copy numbers of β-glucosidases in the recombinant strains were determined by qPCR using extracted genomic DNA as the template (72). All the primers used are described in Table S1.

### Confocal imaging.

Confocal images of recombinant *T. reesei* strains were taken using a confocal SP8 microscope (Leica, Germany) with a ×20 or ×100 oil immersion objective. The excitation wavelength was 552 nm, and the emission wavelength was 570 to 700 nm. ER-Green (KeyGEN BioTECH Co. Ltd., China), GolgiGreen (KeyGEN BioTECH Co. Ltd., China), and GC-PEG-cholesterol-FITC (42) were utilized to stain the ER, Golgi body, and cell wall, respectively, which were carried out as described previously (19). For nuclear staining, fresh mycelia were washed with phosphate-buffered saline (PBS) two times before being suspended in DAPI solution (KeyGEN BioTECH Co. Ltd., China) at 28°C for 10 min. Then, the samples were washed with PBS two times and detected under a confocal microscope with an excitation wavelength of 405 nm and an emission wavelength of 420 to 480 nm.
