# Supplementary figures and images for: Dissecting Cellular Function and Distribution of β-Glucosidases in Trichoderma reesei
Source: mBio. 2021 May 11;12(3):e03671-20. doi: 10.1128/mBio.03671-20 (PMC8262880; doi:10.1128/mBio.03671-20)

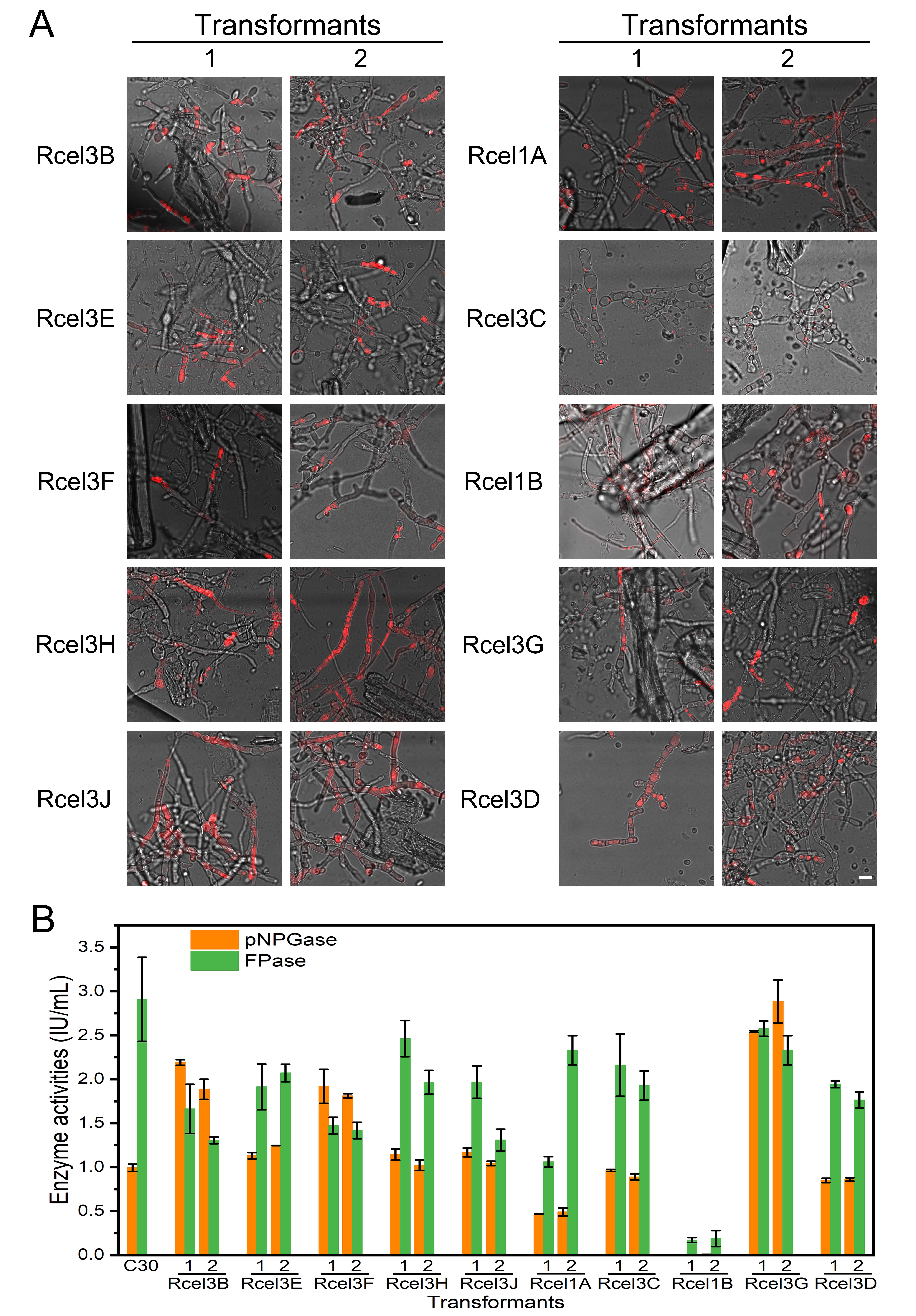

Supplement: FIG S1 [file mbio.03671-20-sf001.jpg]

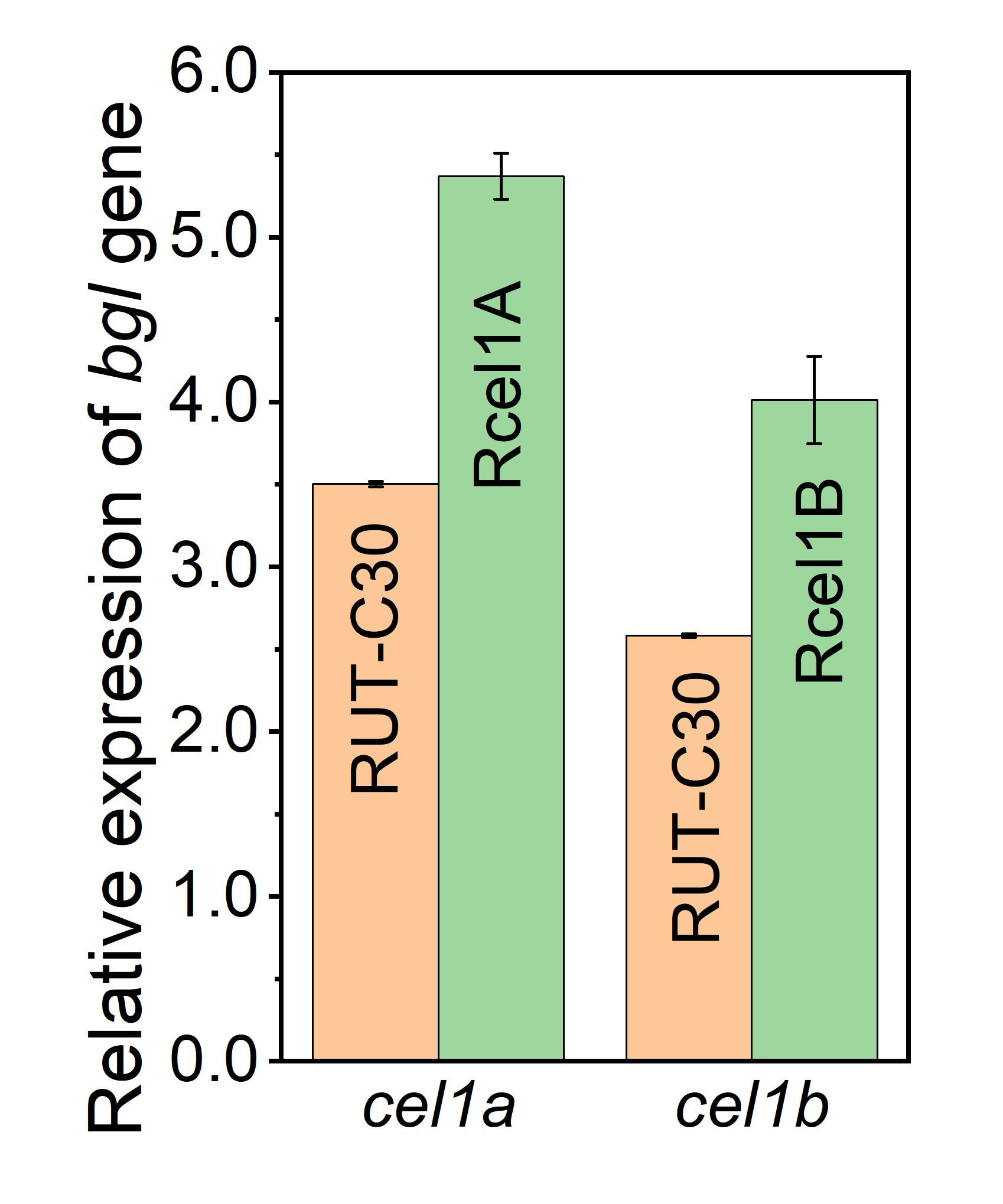

Supplement: FIG S2 [file mbio.03671-20-sf002.jpg]

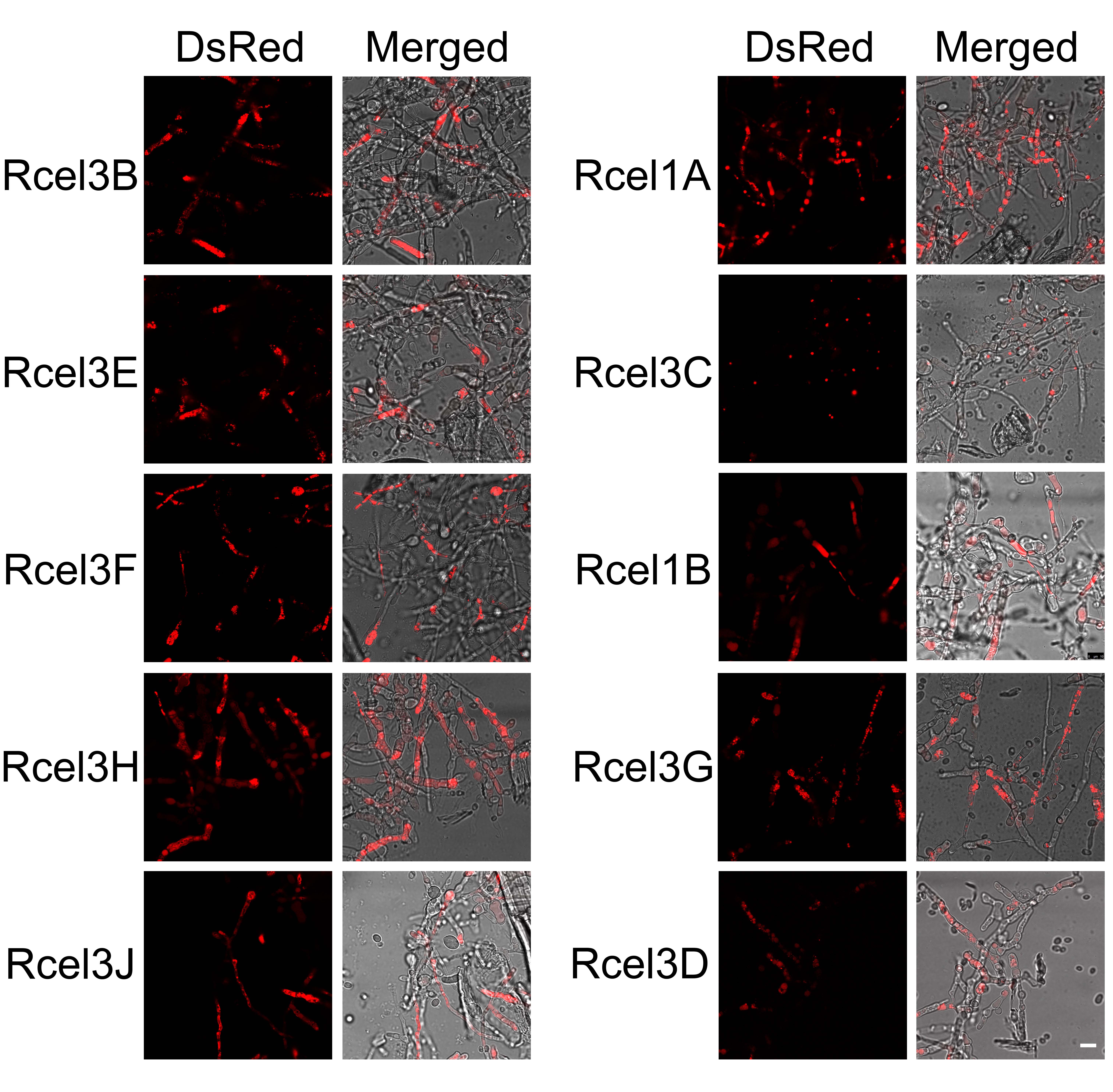

Supplement: FIG S3 [file mbio.03671-20-sf003.jpg]

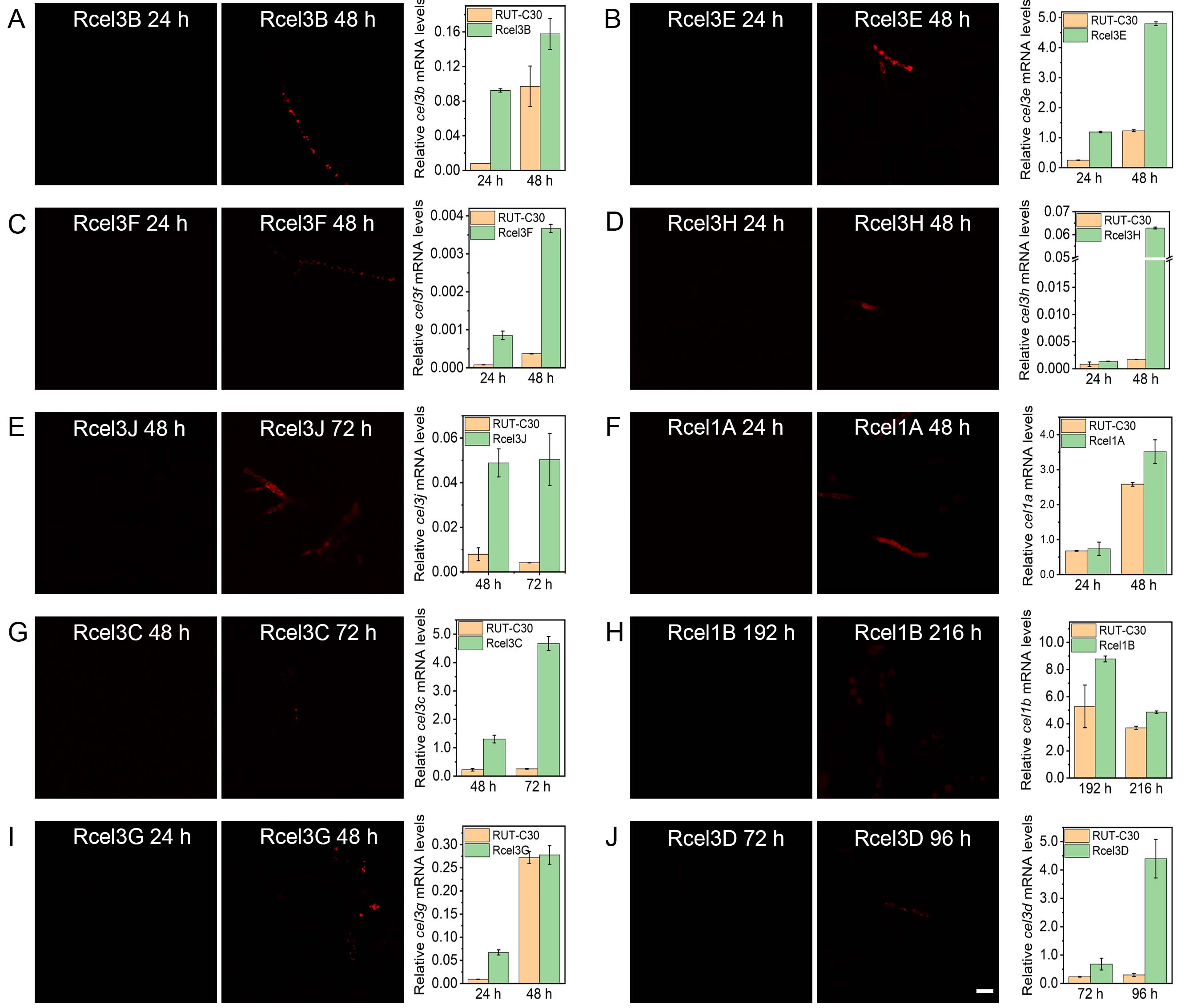

Supplement: FIG S4 [file mbio.03671-20-sf004.jpg]

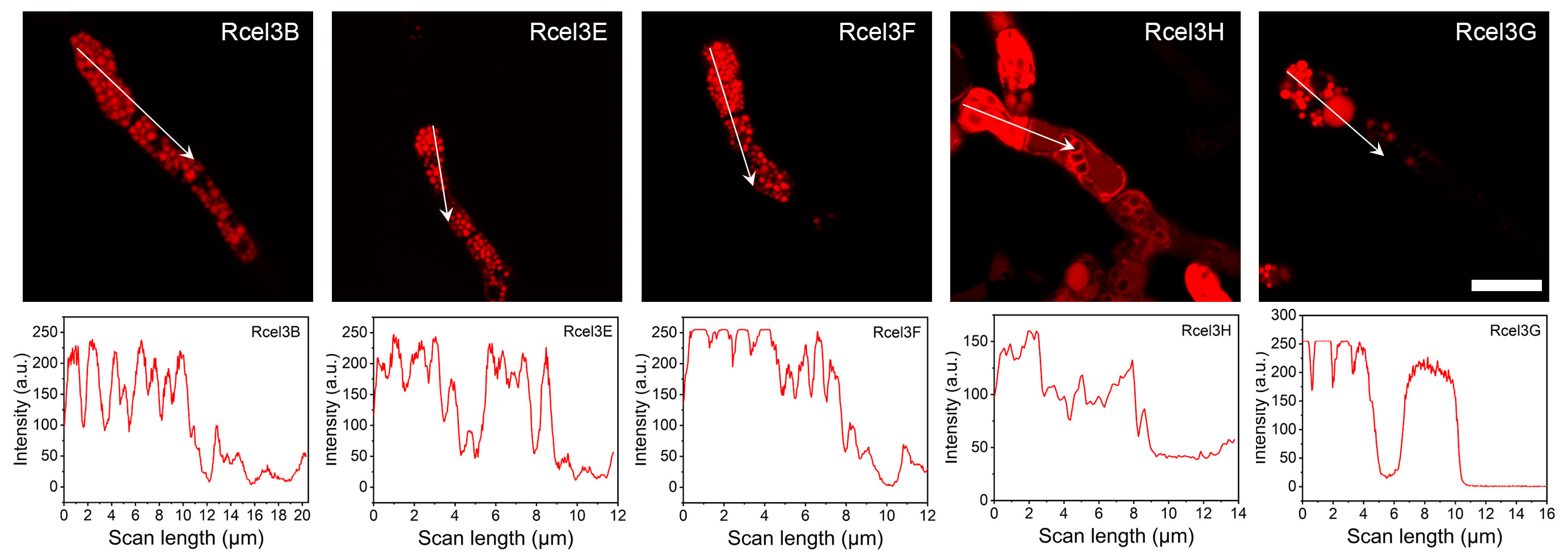

Supplement: FIG S5 [file mbio.03671-20-sf005.jpg]

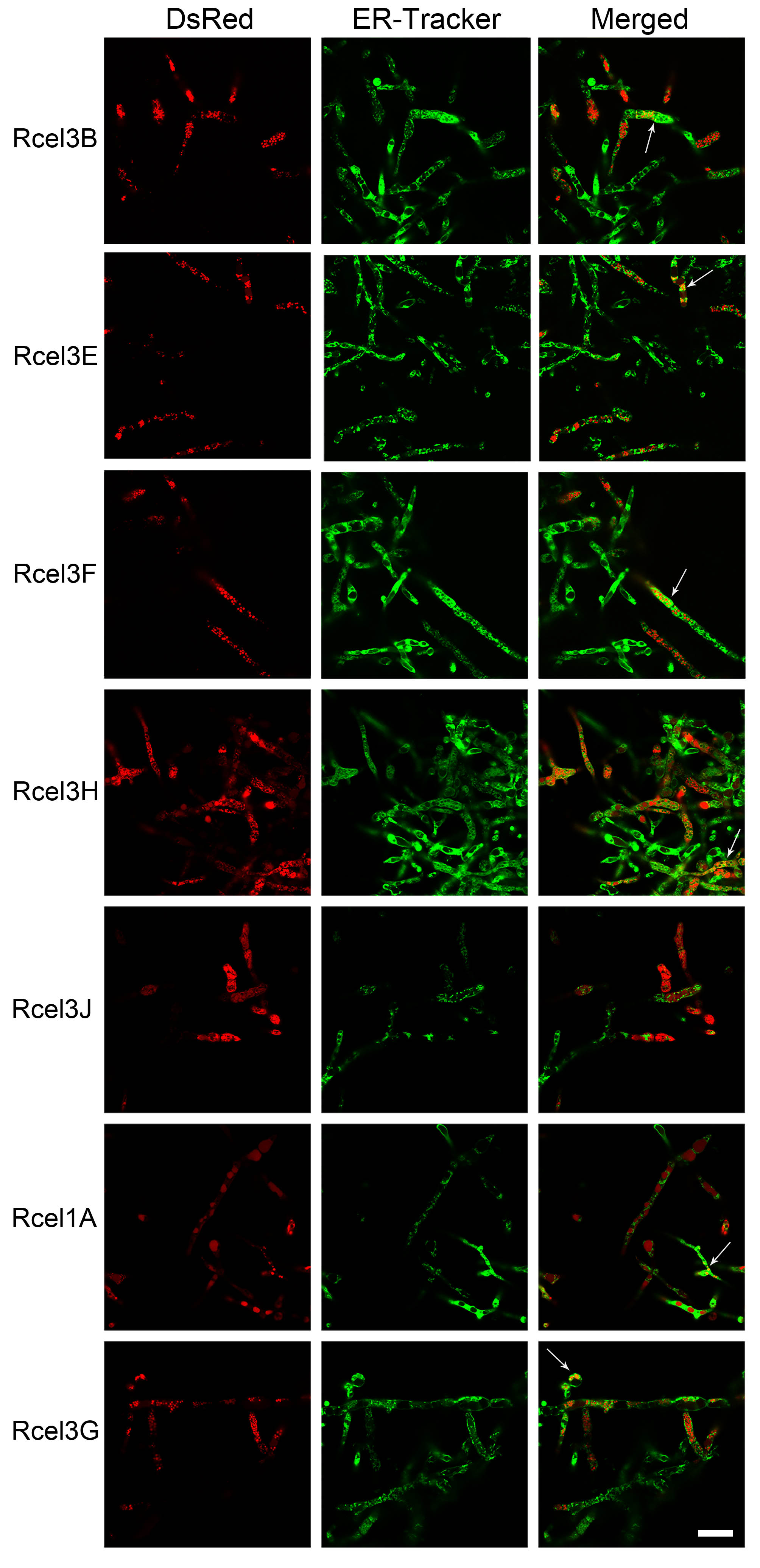

Supplement: FIG S6 [file mbio.03671-20-sf006.jpg]

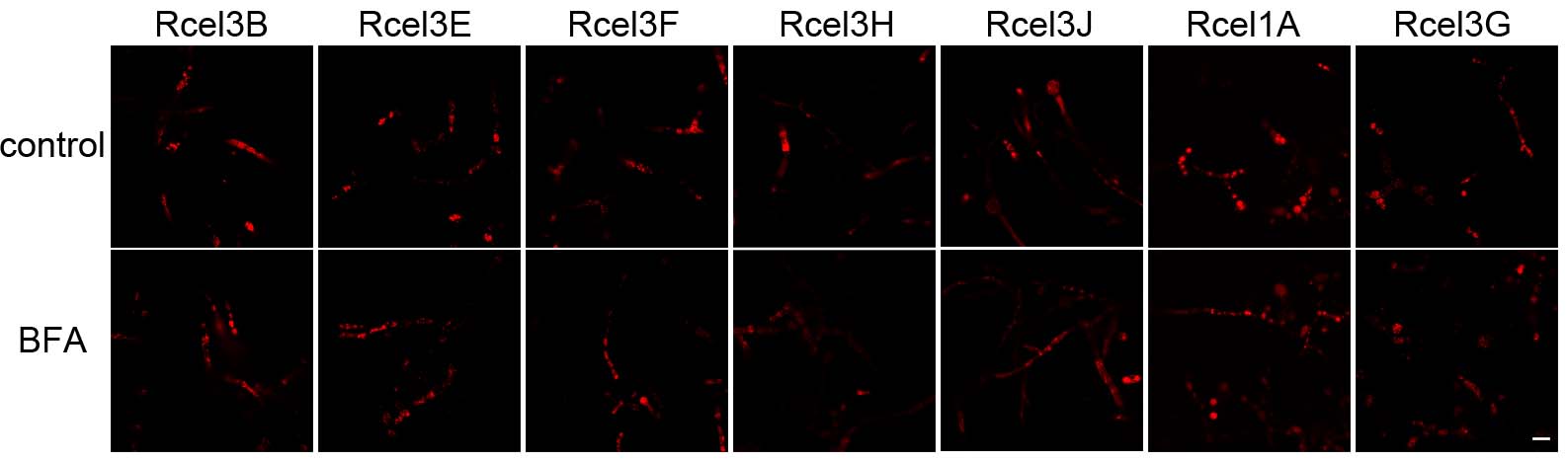

Supplement: FIG S7 [file mbio.03671-20-sf007.jpg]

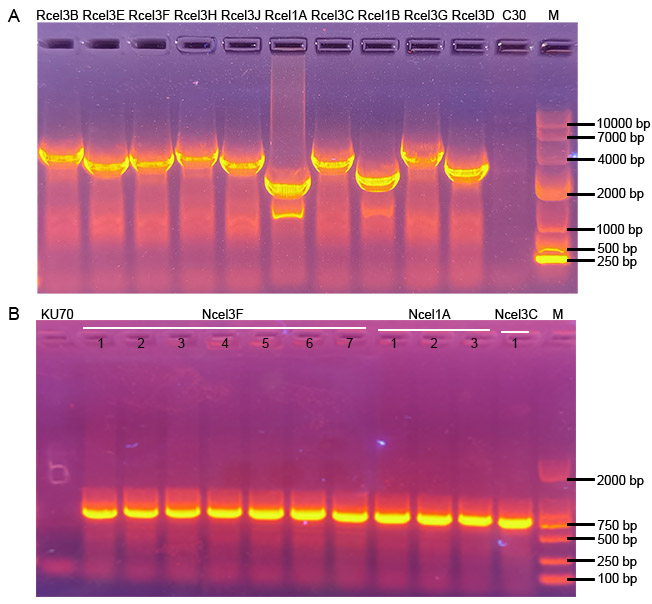

Supplement: FIG S8 [file mbio.03671-20-sf008.jpg]
