# Supplementary material for: Dissecting Cellular Function and Distribution of β-Glucosidases in Trichoderma reesei
Source: mBio. 2021 May 11;12(3):e03671-20. doi: 10.1128/mBio.03671-20 (PMC8262880; doi:10.1128/mBio.03671-20)
Supplement: TABLE S1 [file mbio.03671-20-st001.docx]

Table S1 Primers for β-glucosidase cloning and qPCR.

| Primer | Sequence (5’– 3’) |
| --- | --- |
| **For β-glucosidase overexpression** | |
| Cel1b-dsred F | ACCCAATAGTCAATCTAGAATGCCCGAGTCGCTAGCTCTG |
| Cel1b-dsred R | CTGCTGCTGCCGCTTCTAGATGCCGCCACTTTAACCCTCT |
| Cel3b-dsred F | ACCCAATAGTCAATCTAGAATGAAGACGTTGTCAGTGTTTGCTGCC |
| Cel3b-dsred R | CTGCTGCTGCCGCTTCTAGATGGCAGGCGGGCGCTCAG |
| Cel3c-dsred F | ACCCAATAGTCAATCTAGAATGGCTGATATTGATGTTGAGGC |
| Cel3c-dsred R | CTGCTGCTGCCGCTTCTAGACACGCCAGACCACCAATACGT |
| Cel3e-dsred F | ACCCAATAGTCAATCTAGAATGCGGCTGTGTGACTTATC |
| Cel3e-dsred R | CTGCTGCTGCCGCTTCTAGAAATATGCAGTTTTCCACTGAGTC |
| Cel3f-dsred F | ACCCAATAGTCAATCTAGAATGGTAGCTGTCAAGCAGATTGC |
| Cel3f-dsred R | CTGCTGCTGCCGCTTCTAGAGATGTCCAGCGTCCCATTGA |
| Cel3h-dsred F | ACCCAATAGTCAATCTAGAATGCGGCTCAAACATTGGA |
| Cel3h-dsred R | CTGCTGCTGCCGCTTCTAGAACCAACGCACTGCAGCGT |
| Cel3j-dsred F | ACCCAATAGTCAATCTAGAATGCTGCCTCGCCGGATG |
| Cel3j-dsred R | CTGCTGCTGCCGCTTCTAGACCACGTCCCCGAAACTCG |
| Cel1a-dsred F | ACCCAATAGTCAATCTAGAATGTTGCCCAAGGACTTTCAG |
| Cel1a-dsred R | CTGCTGCTGCCGCTTCTAGACGCCGCCGCAATCAGCTC |
| Cel3g-dsred F | ACCCAATAGTCAATCTAGAATGACCTCGTTTCACGACGGC |
| Cel3g-dsred R | CTGCTGCTGCCGCTTCTAGACACAGGCCCAACAACACCAGT |
| Cel3d-dsred F | ACCCAATAGTCAATCTAGAATGATTCTCGGCTGTGAAAGC |
| Cel3d-dsred R | CTGCTGCTGCCGCTTCTAGACAGCCCCATCCAGAATCG |
|  | |
| **For β-glucosidase expression at its endogenous locus** | |
| 1a-red-up-F | ATTATTATGGAGAAACTCGAGCATAAATGCCACCTCCAGC |
| 1a-red-up-R | CCGAGCCACCGCCACCCTCGAGCGCCGCCGCAATCAGCTCGTCAA |
| 1a-red-do-F | GTGAGGGTTAATTGCGCGGATCCATGGAAAAAGTTTTTGAGTTGATG |
| 1a-red-do-R | CAGGTCGACTCTAGAGAGGATCCCTCTCGTCAACTCAAGTCTTCC |
| 3c-red-up-F | ATTATTATGGAGAAACTCGAGCATGCGTGGCACAGACGGACTCAT |
| 3c-red-up-R | CCGAGCCACCGCCACCCTCGAGCACGCCAGACCACCAATACGTCTC |
| 3c-red-do-F | GTGAGGGTTAATTGCGCGGATCCAAGTCGTGCATCATCTTTGGCAG |
| 3c-red-do-R | CAGGTCGACTCTAGAGAGGATCCCAGAGGCCGATTTCTTGAAGCT |
| 3f-red-up-F | ATTATTATGGAGAAACTCGAGACAACGAAATGCCGCCCCTTCT |
| 3f-red-up-R | CCGAGCCACCGCCACCCTCGAGGATGTCCAGCGTCCCATTGA |
| 3f-red-do-F | GTGAGGGTTAATTGCGCGGATCCAGAGTACTACGGAGGGCAAGAAAATGC |
| 3f-red-do-R | CAGGTCGACTCTAGAGAGGATCCGATGCTGATGACGATGATGAGGTGCTA |
|  | |
| **For PCR confirmation of BGL overexpression in *T. reesei* transformants** | |
| pDht-yz-F | CTCCGGGCAAATGCAAAGTGTGG |
| pDht-yz-R | GTCACCTTCAGCTTCACGGT |
| **For PCR confirmation of BGL expression at its endogenous locus in *T. reesei* transformants** | |
| Rednos-F | CGACGTGAAGCTTATGGCCT |
| Rednos-R | TTAGTGGTGGTGGTGGTGGTGT |
|  | |
| **For qRT-PCR** | |
| Qsar1-F | TGGATCGTCAACTGGTTCTACGA |
| Qsar1-R | GCATGTGTAGCAACGTGGTCTTT |
| Qcel7a-F | GCGGATCCTCTTTCTCAGAC |
| Qcel7a-R | TTGGCGTAGTAATCATCCCA |
| Qcel7b-F | ACTACACGGAGGAGCTCGACGACTT |
| Qcel7b-R | AAGGCATTGCGAGTAGTAGTCGTTG |
| Qcel3a-F | ATGCGTTACCGAACAGCAGCTGC |
| Qcel3a-R | TGCGGCCTTCGCCTTGTCGTAC |
| Qcel1a-F | CCTACCAGATCGAGGGCGC |
| Qcel1a-R | GCAGCGCAATGTCCTCGG |
| Qcel1b-F | TCGCACTTGGACTCGATTTCC |
| Qcel1b-R | CTTGAGGGTGGTGTAGTCTGTGAAC |
| Qcel3b-F | CAACCTCACCACCGGTGTTG |
| Qcel3b-R | AAGCCAACGCTGAAAGCGC |
| Qcel3c-F | CCCTGGCCGAGAAGGTCGA |
| Qcel3c-R | AGCAGGCCGCAGGGACGC |
| Qcel3d-F | GCGAGGATGTTTACGTTGGCTAC |
| Qcel3d-R | TGGGCTGGTCCGGGTGGA |
| Qcel3e-F | TCTTGGGGCAAGAATCTGACAG |
| Qcel3e-R | CTGTTTCAAGCCGATGCCC |
| Qcel3f-F | TCAAGCAGATTGCCCTGCTT |
| Qcel3f-R | GCTTTGGCCTTTTGATACGC |
| Qcel3g-F | GGGTGGAGGATTGGCAAGAG |
| Qcel3g-R | GAAACTCGAAACGCAGAGCC |
| Qcel3h-F | CCGACGATATTGTCGGAGCT |
| Qcel3h-R | GAGAATTGTCCAGCTGGAGC |
| Qcel3j-F | GCAGTCATCGCCATCATCGT |
| Qcel3j-R | TCTCGGCAGCCTTTTGGTAG |
| **For copy number assay** | |
| Nsar-F | CCGGAAAGACCACGTTGCTA |
| Nsar-R | CCTCCTCCCATCGCAGAAAA |
| Ncel1a-F | TCCCTTCAAACGCCCCCC |
| Ncel1a-R | GCAGCGCAATGTCCTCGG |
| Ncel1b-F | TTCTCTGGCAACCCGTTCTC |
| Ncel1b-R | CTTGAGGGTGGTGTAGTCTGTGAAC |
| Ncel3b-F | CAACCTCACCACCGGTGTTG |
| Ncel3b-R | AAGCCAACGCTGAAAGCGC |
| Ncel3c-F | CCCTGGCCGAGAAGGTCGA |
| Ncel3c-R | TCCATGCTTGGGGAGAGCC |
| Ncel3d-F | GCGAGGATGTTTACGTTGGCTAC |
| Ncel3d-R | TGGGCTGGTCCGGGTGGA |
| Ncel3e-F | TCTTGGGGCAAGAATCTGACAG |
| Ncel3e-R | CTGTTTCAAGCCGATGCCC |
| Ncel3f-F | TCAAGCAGATTGCCCTGCTT |
| Ncel3f-R | GCTTTGGCCTTTTGATACGC |
| Ncel3g-F | CTACTGCTGCGTCGGCAAAT |
| Ncel3g-R | GAATCGACCAGAGCCTTGGC |
| Ncel3h-F | AGCTTCTCCTGACAAGGCTC |
| Ncel3h-R | GAGAATTGTCCAGCTGGAGC |
| Ncel3j-F | AGCTTTGACACCACTATCCCC |
| Ncel3j-R | GGATAGCGGTTGCTCTTTGTC |
